# Localization of Generalized Wannier Bases Implies Chern Triviality in Non-periodic Insulators

**DOI:** 10.1007/s00023-022-01232-7

**Published:** 2022-09-25

**Authors:** Giovanna Marcelli, Massimo Moscolari, Gianluca Panati

**Affiliations:** 1 Mathematics Area, SISSA, Via Bonomea 265, 34136 Trieste, Italy; 2 Fachbereich Mathematik, Eberhard Karls Universität Tübingen, Auf der Morgenstelle 10, 72076 Tübingen, Germany; 3 Dipartimento di Matematica, “La Sapienza” Università di Roma, Piazzale Aldo Moro 2, 00185 Rome, Italy

## Abstract

We investigate the relation between the localization of generalized Wannier bases and the topological properties of two-dimensional gapped quantum systems of independent electrons in a disordered background, including magnetic fields, as in the case of Chern insulators and quantum Hall systems. We prove that the existence of a well-localized generalized Wannier basis for the Fermi projection implies the vanishing of the Chern character, which is proportional to the Hall conductivity in the linear response regime. Moreover, we state a localization dichotomy conjecture for general non-periodic gapped quantum systems.

## Introduction

Wannier bases have become, in the last few decades, a fundamental tool in theoretical and computational solid-state physics, as they provide a reasonable compromise between localization in position space and localization in energy, as far as compatible with the uncertainty principle [59]. Whenever it is well localized, a Wannier basis:

(i) allows to implement numerical algorithms whose computational costs scale only linearly with the system size [33];
(ii) provides a key tool for a simple and transparent description of macroscopic polarization and orbital magnetization in solids, yielding to computable formulae [16, 43], later proved in a broader setting by more advanced mathematical techniques [74, 76];
(iii) allows an efficient numerical treatment of deformed periodic systems [83];
(iv) helps to justify the so-called atomic limit, *i. e. *, the description of macroscopic solids as “consisting of well-localized atoms”, a classical paradigm whose violation has opened the new field of topological chemistry [11].

Last but not least, the variational characterization of Wannier functions proposed by Marzari and Vanderbilt has turned Wannier bases into an efficient and flexible computational tool [59, 60, 73].

Since these advantages rely on good decay properties of Wannier functions, several works have been dedicated to the rigorous proof of the existence of an exponentially localized Wannier basis in periodic (time-reversal symmetric) insulators. These research efforts started with the pioneering work of Kohn and des Cloizeaux [27, 28, 45] and continued with the work of Nenciu and Helffer-Sjöstrand [38, 67–69], till the modern bundle-theoretic methods [13, 72]. More recently, the emphasis has shifted from abstract to *algorithmic* proofs of existence [19, 20, 22, 23, 31], which allow a direct numerical implementation [15]. Neglecting the linear independence condition, the related concept of *Parseval frame* has also been investigated [2, 22, 47].

When the Hamiltonian breaks time-reversal symmetry (TRS), as, for example, in Chern insulators and quantum Hall systems, the existence issue becomes more involved and interesting. A *Localization–Topology Correspondence*, also dubbed *Localization Dichotomy*, has been noticed and proved in [62]. There the authors proved that in a gapped $\Gamma$-periodic insulator in dimension $d \le 3$, with $\Gamma \simeq \mathbb {Z}^d$ a Bravais lattice, a Wannier basis $w = \left\{ w_{\gamma , a} \right\} _{\gamma \in \Gamma , 1 \le a \le m}$ which is well localized, in the sense that there exists $M_{*}<\infty$ such that

$$\begin{aligned} \langle X^2\rangle _w := \sum _{a=1}^m \int _{\mathbb {R}^d} |\mathbf{x}|^2 \, |w_{\gamma , a}(\mathbf{x}+ \gamma )|^2 \mathrm{d}\mathbf{x}\le M_* \qquad \forall \gamma \in \Gamma , \end{aligned}$$

exists if and only if the Bloch bundle associated with the Fermi projection *P* is Chern trivial. In $d=2$, the latter condition is equivalent to the vanishing of the (first) *Chern number*, defined by

1.1 $$\begin{aligned} c_1(P) = \frac{1}{2\pi } \int _{\mathbb {T}^2_*} {{\,\mathrm{Tr}\,}}\Big ( P(\mathbf{k}) \big [ \partial _{k_1}P(\mathbf{k}) , \partial _{k_2}P(\mathbf{k}) \big ] \Big ) \, \mathrm{d}k_1 \wedge \mathrm{d}k_2, \end{aligned}$$

where $\mathbb {T}^2_* = \mathbb {R}^2/\Gamma ^*$ is the two-dimensional Brillouin torus and $\mathcal {U}_{\mathrm {BF}} \, P \, \mathcal {U}_{\mathrm {BF}}^{-1}= \int ^{\oplus }_{\mathbb {T}^2_*} P(\mathbf{k}) \, d\mathbf{k}$ is the Bloch–Floquet–Zak decomposition of *P*; see, *e.g.*, [48] or [61].

For $d=3$, the Chern triviality corresponds to the vanishing of three “first Chern numbers” defined, for $i \ne j \in \left\{ 1,2,3 \right\}$, by^1^

1.2 $$\begin{aligned} c_1(P)_{ij} = \frac{1}{2\pi } \int _{\mathbb {B}_{ij}} {{\,\mathrm{Tr}\,}}\Big ( P(\mathbf{k}) \big [ \partial _{k_i}P(\mathbf{k}) , \partial _{k_j} P(\mathbf{k}) \big ] \Big ) \, \mathrm{d}k_i \wedge \mathrm{d}k_j, \end{aligned}$$

where $\mathbb {B}_{ij} \subset \mathbb {T}^3_*$ is the two-dimensional subtorus of the Brillouin torus $\mathbb {T}^3_* = \mathbb {R}^3/\Gamma ^*$ obtained by fixing the coordinate different from the *i*-th and the *j*-th (*e.g.*, equal to zero). Moreover, if the Bloch bundle is Chern trivial, then an exponentially localized Wannier basis always exists for $d\le 3$ [13, 72].

To avoid any source of confusion, we emphasize here that the Localization–Topology Correspondence (LTC) refers to a different kind of localization mechanism than the one appearing in the well-known Anderson localization. Moreover, while Anderson localization concerns the decay properties of *eigenfunctions* of Schrödinger operators with random potentials, the LTC refers to the decay properties of particular orthornormal bases, namely generalized Wannier bases, that span the spectral subspaces of a (possibly non-periodic) Hamiltonian operator.

This paper aims at the generalization of the LTC from the periodic setting considered in [62] to the non-periodic one. Since both sides of the correspondence, namely Wannier bases and Chern numbers, are defined by using periodicity in an essential way, even the formulation of a reasonable conjecture requires some care.

On the side of Wannier bases, a generalization of this concept to non-periodic systems has been discussed since the early work of Kohn and Onffroy [46]. Later, Kivelson noticed that for $d=1$ a generalized Wannier basis is provided by the eigenfunctions of the reduced position operator ${\widetilde{X}} := PXP$, where *X* is the usual position operator [44]. This intuition has been put on solid mathematical grounds in [71], where it is proved that for any gapped one-dimensional Schrödinger operator the spectrum of ${\widetilde{X}}$ is discrete, and the corresponding eigenfunctions form a ***generalized Wannier basis*** (GWB)^2^, as reviewed in Example 2.7. While the above construction does not generalize to $d>1$, the existence of a GWB can be proved for several specific *d*-dimensional systems, as discussed in Sect. 2.1 following [70] and [10]. Moreover, in [26] it has been shown that the construction of [71] is optimal, in the sense that the obtained GWB has the same exponential decay of the associated Fermi projection. An alternative strategy to construct a GWB for a generic two-dimensional non-periodic system has been recently suggested and numerically validated [79]. We shortly review the whole topic of GWB in Sect. 2.1.

On the other side of the correspondence, for $d=2$ the Chern number of the Bloch bundle is naturally generalized to non-periodic models by the ***Chern character***, defined, for a sufficiently regular orthogonal projection *P* acting on $L^2(\mathbb {R}^2)$, by

1.3 $$\begin{aligned} C(P) := 2\pi \,\, \tau \Big ( \mathrm {i}P \big [ [X_1, P], [X_2, P] \big ] \Big ) \end{aligned}$$

where $\tau (\cdot )$ is the trace per unit volume. Whenever *P* is periodic the above formula reduces to (1.1), so that $C(P)=c_1(P)$. Physically, $\frac{1}{2\pi }C(P)$ gives in Hartree units^3^ the Hall conductivity of the system, which agrees with the Hall conductance under mild technical assumptions [5].

In connection with solid-state physics, formula (1.3) first appeared—to the best of our knowledge—in 1986 in a conference proceedings by J. Bellissard [6], where *C*(*P*) is baptized Chern character and it is specified that formula (1.3) applies to orthogonal projectors affiliated to a specific $C^*$-algebra of *ergodic* operators (reducing to *periodic* operators in the deterministic case). However, as early envisaged by Bellissard himself, the same formula makes sense in a broader context, for projectors *P* whose kernel is sufficiently fast decreasing away from the diagonal [66]. In an ergodic setting, this viewpoint and its relation with non-commutative geometry have been deeply explored in [7]. The relation with the index of a pair of projections and with the Fredholm index has been also clarified [5, 6, 42], see the review paper [34] and references therein.

The same formula (1.3) has later been reconsidered in a non-ergodic and non-covariant setting, provided the trace per unit volume exists, which it happens in particular for exponentially localized projections, as in Definition 2.3. Interpreted in this broader sense, formula (1.3) still produces an integer whenever the trace per unit volume exists, as proved in [29] for discrete models. The latter proof, which is essentially based on the identity between the Chern character and the index of a Fredholm operator as previously established in the covariant and ergodic setting [5, 42], is generalized to gapped continuum models in Proposition 2.13.

Finally, in 2011 physicists rediscovered an equivalent version of formula (1.3), namely $C(P) = 2\pi \mathrm {i}\, \tau ( [ P X_1 P, P X_2 P])$, labeling the corresponding quantity as *Chern invariant* [8] and *Chern marker* [14, 40]. The latter name has been also used in the recent mathematical literature [21, 55].

In this paper, we conjecture a Localization–Topology Correspondence for non-periodic gapped systems (Conjecture 3.2) and we prove one of the conjectured implications, with a non-optimal threshold (Theorem 3.1): if an orthogonal projection *P*, which acts on $L^2(\mathbb {R}^2)$ and is exponentially localized in the sense of Definition 2.3, admits a GWB $\{\psi _{\gamma ,a}\}_{\gamma \in \mathfrak {D}, 1 \le a \le m(\gamma )}$ (here $\mathfrak {D}$ is a discrete set, as in Definition 2.5) which is *s*-localized for some $s>4$, *i. e. *, for $s>4$ there exists $M<+\infty$ such that

$$\begin{aligned} \int _{\mathbb {R}^2} \langle \mathbf{x}- \gamma \rangle ^{2s} \,\, |\psi _{\gamma ,a}(\mathbf{x})|^2 \,\mathrm{d}\mathbf{x}\le M \qquad \forall \gamma \in \mathfrak {D},\,1 \le a \le m(\gamma ), \end{aligned}$$

then the corresponding Chern character *C*(*P*) vanishes. Notice that neither periodicity nor covariance with respect to the action of a group is required. The fact that our result does not assume periodicity or covariance makes it suitable for applications also to random Schrödinger operators [1]. In that context, it has been proved that localized eigenfunctions, whose localization might be caused by very different mechanisms, do not contribute to the conductivity: The case of localization of eigenfunctions due to deep wells has been analyzed in [66], while the case of Anderson localization has been treated in [32]; see also the earlier work [49]. Although Wannier functions are not eigenfunctions, our results say—coherently with the latter papers—that the existence of a well-localized GWB implies the vanishing of the transverse charge conductivity.

### Further References

We conclude the Introduction mentioning some results appeared during the revision of our manuscript. The result in this paper was first announced in [55], and a preliminary version of the proof was provided in the PhD thesis of one of the authors [64]. These preliminary papers, together with the results in [62], resparked the interest of part of the community for the analysis of Wannier bases for non-periodic systems. Besides the aforementioned [79, 80], we notice the preprint by Lu and Stubbs [52] (see also [51]) where they manage to show Theorem 3.1 with $s>1$. Nevertheless, Theorem 3.1 with the optimal threshold $s\ge 1$ is still an open problem. Finally, it is worth to notice that the LTC has been recently generalized also in a different direction, within the $C^*$-algebraic approach to solid-state physics [9, 53].

## Setting and Fundamental Concepts

As explained in the Introduction, the aim of this paper is to generalize results from the periodic setting to the non-periodic one. In order to model materials that are not exactly crystalline we have to replace the *Bravais lattice*^4^, which models the periodicity of a crystalline system, by a discrete set $\mathfrak {D}$. We require some uniformity as in the following definition, where $B_\rho (\mathbf{x})\subset \mathbb {R}^d$ denotes the open ball of radius $\rho >0$ centered in $\mathbf{x}\in \mathbb {R}^d$.

### Definition 2.1

A set $\mathfrak {D}\subset \mathbb {R}^d$ is said to be *r*-*uniformly discrete* if there exists $r>0$ such that $\forall \,\mathbf{x}\in \mathbb {R}^d$ the set $B_{r}(\mathbf{x}) \cap \mathfrak {D}$ contains at most one element.

Obviously, a Bravais lattice $\Gamma \simeq \mathbb {Z}^d$ is a *r*-uniformly discrete set for a suitable $r>0$.

A central role in our analysis will be played by the concept of localization in space; hence, the following Definition will be useful.

### Definition 2.2

*(Localization function).* We say that a continuous function

$$\begin{aligned} G:[0,\infty )\rightarrow (0,\infty ) \end{aligned}$$

is a *localization function* if $\lim _{x \rightarrow \infty }G(x)=+\infty$ and there exists a constant $C_G>0$ such that

2.1 $$\begin{aligned} G(\left\| \mathbf{x}-\mathbf{y} \right\| )\le C_G \,G(\left\| \mathbf{x}-\mathbf{z} \right\| )G(\left\| \mathbf{z}-\mathbf{y} \right\| )\qquad \forall \,\mathbf{x},\mathbf{y},\mathbf{z}\in \mathbb {R}^d. \end{aligned}$$

Natural examples of localization functions are $G(x) = {{\,\mathrm{e}\,}}^{\alpha x}$ for some $\alpha > 0$, and $G(x)=\langle x \rangle ^{2s}$ for some $s > 0$, where $\langle x \rangle := \left( 1+ x^2 \right) ^{\frac{1}{2}}$ as usual.

Our aim is to investigate the relation between localization of GWBs and transport properties in non-interacting gapped quantum systems, whose dynamics is generated by a one-particle (magnetic) Schrödinger operator [3]. The spectral projections onto an isolated component of the spectrum of such operators provide archetypal examples of exponentially localized projections, defined as follows.

### Definition 2.3

*(Exponentially localized projection).* We say that an orthogonal projection *P* acting on $L^2(\mathbb {R}^d)$ is *exponentially localized* if *P* is an integral operator with a *jointly continuous* integral kernel $P(\cdot \,,\,\cdot ):\mathbb {R}^d\times \mathbb {R}^d \rightarrow \mathbb {C}$ and there exist two constants $C,\beta >0$ such that

2.2 $$\begin{aligned} \left| P(\mathbf{x},\mathbf{y})\right| \le C \mathrm {e}^{-\beta \Vert \mathbf{x}-\mathbf{y}\Vert }\,\qquad \forall \, \mathbf{x},\mathbf{y}\in \mathbb {R}^d. \end{aligned}$$

The following proposition provides examples of exponentially localized projection for a large class of two-dimensional quantum systems, including magnetic Schrödinger operators with constant magnetic field.

### Proposition 2.4

Let $V: \mathbb {R}^2 \rightarrow \mathbb {R}$ be in $L^2_{\mathrm{uloc}}(\mathbb {R}^2)$, which means that *V* is uniformly locally square-integrable, *i. e. *,

2.3 $$\begin{aligned} \sup _{\mathbf{x}\in \mathbb {R}^2} \int \limits _{\Vert \mathbf{x}-\mathbf{y}\Vert \le 1} |V (\mathbf{y})|^2 \mathrm{d}\mathbf{y}\, < \, \infty . \end{aligned}$$

Assume that the magnetic vector potential $\mathbf{A}: \mathbb {R}^2 \rightarrow \mathbb {R}^2$ is in $L^4_{\mathrm{loc}}(\mathbb {R}^2,\mathbb {R}^2)$ with distributional derivative $\nabla \cdot \mathbf{A} \in L^2_{\mathrm{loc}}(\mathbb {R}^2)$. Consider the Hamiltonian operator

$$\begin{aligned} H_\mathbf{A}:=- \frac{1}{2} \Delta _\mathbf{A} + V \, , \end{aligned}$$

where $$-\Delta _\mathbf{A}:=\left( -\mathrm {i}\nabla - \mathbf {A}\right) ^2$$. Then,

(i) $H_\mathbf{A}$ is essentially self-adjoint on $C^\infty _{\mathrm{c}}(\mathbb {R}^2)$. We denote its closure again by $H_\mathbf{A}$.
(ii) The domain $\mathcal {D}_\mathbf{A}$ of self-adjointness of $H_\mathbf{A}$ is contained in $C(\mathbb {R}^2)$.
(iii) $H_\mathbf{A}$ is bounded from below.

Moreover, assume that $\sigma (H_\mathbf{A})$, the spectrum of $H_\mathbf{A}$, has an *isolated component*, *i. e. *, there exist two non empty sets $\sigma _0,\sigma _1 \subset \mathbb {R}$ and $E_{\pm } \in \mathbb {R}\setminus \sigma (H_\mathbf{A})$ such that

2.4 $$\begin{aligned} \sigma (H_\mathbf{A})=\sigma _0 \cup \sigma _1, \qquad \sigma _0 \subset \left( E_-,E_+\right) \, \text { and }\, \left( E_-,E_+\right) \cap \sigma (H_\mathbf{A})= \sigma _0.\nonumber \\ \end{aligned}$$

Let $P_0$ be the spectral projection corresponding to $\sigma _0$. Then,

(iv) $P_0$ is an exponentially localized projection in the sense of Definition 2.3.

We refer to $P_0$ as the *Fermi projection*, interpreting it as the projection onto the space of “occupied” states of a system of non-interacting particles.

### Sketch of the proof

The statements (i) and (iii) are a special case of [50, Theorem 3]. The property (ii) is a consequence of the fact that the Schrödinger semigroup $e^{-t H_A}$ is a bounded operator from $L^2(\mathbb {R}^2)$ to $L^\infty (\mathbb {R}^2)$ [12, equation (2.40)] and maps $L^2(\mathbb {R}^2)$ into the space of continuous functions [12, Theorem 4.1]. Therefore, repeating the proof of [77, Corollary B.3.2, Theorem B.3.3] one can show that, for $\lambda >0$ large enough, the resolvent $\left( H_\mathbf{A} + \lambda \right) ^{-1}$ maps $L^2(\mathbb {R}^2)$ into the space of continuous functions. Regarding the statement (iv), the existence and joint continuity of the integral kernel of $P_0$ is a standard result in the theory of Schrödinger operators [12, Remarks 6.2.(ii)] [77]. The exponential localization of the integral kernel is a consequence of the Combes–Thomas estimates on the resolvent [17], which can be shown by adapting the proofs of [24, Proposition 3.1, Appendix A], coupled with the spectral gap assumption on $\sigma _0$, which allows to choose an integration contour $\mathcal {C}\subset \mathbb {C}$ with a uniform positive distance from $\sigma _0$, and the fact that $P_0 =-\frac{\mathrm {i}}{2 \pi } \oint _{\mathcal {C}} dz \, z (H_\mathbf{A}-z)^{-2}$; see [64, Appendix A.1.1] for details. $\square$

### Generalized Wannier Bases: A Short Review

Following the seminal idea in [70, 71] we define generalized Wannier bases and functions as follows.

#### Definition 2.5

*(Generalized Wannier basis).* Let *P* be an orthogonal projection acting on $L^2(\mathbb {R}^d)$ . We say that *P* admits a *generalized Wannier basis* (GWB) if there exist a localization function $G$, a discrete set $\mathfrak {D}\subset \mathbb {R}^d$, a constant $m_*>0$ and a set $\{\psi _{\gamma ,a}\}_{\gamma \in \mathfrak {D}, 1 \le a \le m(\gamma )}\subset L^2(\mathbb {R}^d)$ with $m(\gamma )\le m_*$ for every $\gamma \in \mathfrak {D}$, such that:

(i) $\{\psi _{\gamma ,a}\}_{\gamma \in \mathfrak {D}, 1 \le a \le m(\gamma )}$ is an orthonormal basis for ${{\,\mathrm{Ran}\,}}P$;
(ii) there exists $M<\infty$ such that every $\psi _{\gamma ,a}$ is $G$-*localized around* $\gamma$, *i. e. *,
  2.5 $$\begin{aligned} \int _{\mathbb {R}^d} |\psi _{\gamma ,a}(\mathbf{x})|^2 G(\Vert \mathbf{x}-\gamma \Vert ) \, \mathrm{d}\mathbf{x}\le M \quad \forall \gamma \in \mathfrak {D},\,1 \le a \le m(\gamma ). \end{aligned}$$

If the above conditions are satisfied, we say that *P* admits a GWB which is $G$-*localized around the set* $\mathfrak {D}$. One also says that $\psi _{\gamma ,a}$ is a *generalized Wannier function* (GWF) $G$-localized around $\gamma \in \mathfrak {D}$.

Notice that the localization center $\gamma \in \mathfrak {D}$ is far from being unique, even in the periodic case. In the physics literature, the vector $\langle \psi _{\gamma ,a}, \mathbf {X} \psi _{\gamma ,a}\rangle$ is called *centroid* of $\psi _{\gamma ,a}$. Moreover, in contrast with the usual definition of Wannier functions for periodic systems, the index $m(\gamma )$ might depend on $\gamma \in {\mathfrak {D}}$. Indeed, without a periodicity assumption, one may in general expect a different number of orbitals in different lattice sites. The only constraint we require is that such a number is uniformly bounded, namely $m(\gamma ) \le m_*, \, \forall \gamma \in {\mathfrak {D}}$.

Finally, it might seem natural to require, in the definition of GWB, that the set $\mathfrak {D}$ is a *Delone set*, *i. e. *, both uniformly discrete and uniformly nowhere sparse^5^. However, as we do not use both these properties in our Theorem (only uniform discreteness is assumed), we preferred not to include them in the definition.

The following terminology agrees with [62]:

- $\diamond$ if (2.5) holds true with $G(\Vert \mathbf{x}\Vert )={\mathrm {e}}^{2 \alpha \Vert \mathbf{x}\Vert }$, for some $\alpha > 0$, we say that the GWB is *exponentially localized*;
- $\diamond$ if (2.5) holds true with $G(\Vert \mathbf{x}\Vert )=\langle \mathbf{x}\rangle ^{2s}$ for some $s > 0$, we say that the GWB is *s-localized*.

In the case of an exponentially localized projection *P*, the $L^2$-localization estimate (2.5) implies a $L^\infty$-estimate on any GWF $\psi _{\gamma ,{a}}$, as in the following

#### Lemma 2.6

(From $L^2$-estimates to $L^\infty$-estimates). Let *P* be an exponentially localized projection acting on $L^2(\mathbb {R}^d)$. Assume that *P* admits a GWB $\{\psi _{\gamma ,a}\}_{\gamma \in \mathfrak {D}, 1 \le a \le m(\gamma )}$ with localization function $G(\Vert \mathbf{x}\Vert ) \le C_1 \mathrm {e}^{\lambda \Vert \mathbf{x}\Vert }$, with $C_1 > 0$ and $\lambda < 2\beta$ for $\beta$ as in (2.2). Then, there exists a constant $K>0$, independent of $\gamma \in \mathfrak {D}$, such that each GWF $\psi _{\gamma ,{a}}$ satisfies

2.6 $$\begin{aligned} \left| \psi _{\gamma ,{a}} (\mathbf{x}) \right| \le K \, G(\Vert \mathbf{x}-\gamma \Vert )^{-1/2} \quad \forall \, \mathbf{x}\in \mathbb {R}^d, \,\, \forall \, \gamma \in \mathfrak {D}. \end{aligned}$$

#### Proof

Using the fact that $\psi _{\gamma ,{a}}=P\psi _{\gamma ,{a}}$, one has

$$\begin{aligned} \left| G(\Vert \mathbf{x}-\gamma \Vert )^{1/2} \psi _{\gamma ,{a}} (\mathbf{x}) \right| \le&\displaystyle \int _{\mathbb {R}^d} \mathrm{d} \mathbf{y}\, G(\Vert \mathbf{x}-\gamma \Vert )^{1/2} \, \left| P(\mathbf{x},\mathbf{y}) \right| \left| \psi _{\gamma ,{a}}(\mathbf{y})\right| \\ \le&C_G^{1/2}\left( \displaystyle \int _{\mathbb {R}^d} \mathrm{d} \mathbf{y}\, G(\Vert \mathbf{x}-\mathbf{y}\Vert ) \, \left| P(\mathbf{x},\mathbf{y}) \right| ^2 \right) ^{1/2} \\&\left( \displaystyle \int _{\mathbb {R}^d} \mathrm{d}\mathbf{y}\,G(\Vert \gamma -\mathbf{y}\Vert ) \,\left| \psi _{\gamma ,{a}}(\mathbf{y})\right| ^2 \right) ^{1/2}, \end{aligned}$$

where in the second and third inequality we have used property (2.1) and the Cauchy–Schwarz inequality, respectively. In view of (2.2) and of the hypothesis on *G*, there exists a constant $K>0$, independent of $\gamma \in \mathfrak {D}$, such that

$$\begin{aligned}&C^{1/2}_G \sup _{\mathbf{x}\in \mathbb {R}^d} \left( \int _{\mathbb {R}^d} \mathrm{d} \mathbf{y}\, G(\Vert \mathbf{x}-\mathbf{y}\Vert ) \, \left| P(\mathbf{x},\mathbf{y}) \right| ^2 \right) ^{1/2} \\&\quad \quad \left( \int _{\mathbb {R}^d} \mathrm{d} \mathbf{y}\, G(\Vert \gamma -\mathbf{y}\Vert ) \,\left| \psi _{\gamma ,{a}}(\mathbf{y})\right| ^2 \right) ^{1/2} \le K\,. \end{aligned}$$

Therefore, (2.6) is proved. $\square$

Whenever the projection *P* admits a GWB, it can be written as (using the Dirac notation)

2.7 $$\begin{aligned} P\;=\; \sum _{\gamma \in \mathfrak {D}} \sum _{1\le a \le m(\gamma )} \left| \psi _{\gamma ,a} \right\rangle \left\langle \psi _{\gamma ,a} \right| \end{aligned}$$

where the series is understood as the strong limit of the finite sums of the projections on the one-dimensional spaces spanned by each GWF. Notice that the strong limit is independent of the ordering of the series. Moreover, if the set $\mathfrak {D}$ is *r*-uniformly discrete, it is convenient to rearrange the above series as

$$\begin{aligned} P \;\varphi =\;\lim _{L\rightarrow \infty } \sum _{\gamma \in \mathfrak {D}\cap \Lambda _L} \sum _{1\le a \le m(\gamma )} \left\langle \psi _{\gamma ,a} \right| \varphi \rangle \psi _{\gamma ,a},\quad \forall \,\varphi \in L^2(\mathbb {R}^d), \end{aligned}$$

with $\Lambda _L = [-L,L]^d$. Furthermore, if $P= P_0$ is the Fermi projection of an operator satisfying the hypotheses of Proposition 2.4 and admitting a GWB that is exponentially localized or *s*-localized, with $s>1$, the equality

2.8 $$\begin{aligned} P_0(\mathbf{x},\mathbf{y})\;=\; \sum _{\gamma \in \mathfrak {D}} \sum _{1\le a \le m(\gamma )} \psi _{\gamma ,a}(\mathbf{x}) \overline{\psi _{\gamma ,a}(\mathbf{y})}\, , \qquad \forall \, \mathbf{x},\mathbf{y}\in \mathbb {R}^2 , \end{aligned}$$

holds true pointwise, since $\psi _{\gamma ,a}\in {{\,\mathrm{Ran}\,}}P_0\subset \mathcal {D}_\mathbf{A}\subset C(\mathbb {R}^2)$ by Proposition 2.4(ii) and $\psi _{\gamma ,{a}}$ satisfies (2.6).

We emphasize a crucial point: The fact that $P_0$ is an exponentially localized projection does *not* imply that the GWFs appearing in (2.8) are themselves exponentially localized, as it is explained in [62] for the periodic case. While the exponential localization of $P_0$ is a mere consequence of the gap condition, via Combes–Thomas estimates, the existence of an exponentially localized Wannier basis is related—in the periodic case—to the Chern triviality of the vector bundle associated with $P_0$ [13, 19, 22, 31, 48, 72]. Notice, moreover, that not every orthonormal basis is able to capture the topological properties of the former vector bundle. Indeed, if one relaxes the definition of GWB, allowing, for example, an orthonormal basis whose elements are localized around circles of increasing radii as in [75], it is always possible to find an exponentially localized orthonormal basis such that (2.8) holds true; see [65]. In this sense, the definition of GWB is a good compromise between generality and ability to “read” the vanishing of the Chern character.

Whenever the Hamiltonian operator is $\Gamma$-periodic and satisfies suitable regularity conditions, see, *e.g.*, [62, Remark 3.2], the Fermi projection $P_0$ commutes with a unitary representation of the group $\Gamma \simeq \mathbb {Z}^d$, denoted by $\left\{ T_\gamma \right\} _{\gamma \in \Gamma }$, and one constructs via Bloch–Floquet theory the usual *composite Wannier basis* for $P_0$, denoted by $\{T_\gamma w_{0,a} \}_{\gamma \in \Gamma , 1 \le a \le m}$, for some fixed integer $m \ge 1$ (equal to the number of Bloch bands associated with $P_0$). It is easy to see that such composite Wannier basis satisfies Definition 2.5, so it is indeed a GWB. According to the value of the first Chern number(s) of *P*, such a GWB can be chosen exponentially localized if $c_1(P) = 0$, or *s*-localized for any $s <1$ (but not for $s=1$!) if instead $c_1(P) \ne 0$ [62].

It is of relevance to note that there are non-periodic systems in which is not possible to construct a composite Wannier basis—in view of the lack of periodicity—but is still possible to have a generalized Wannier basis. In this direction, we review here some examples^6^:

#### Example 2.7

*(Generic one-dimensional insulators).* Consider the one-dimensional Schrödinger operator

2.9 $$\begin{aligned} H=-\frac{\mathrm d^2}{{\mathrm d} x^2} + V \qquad \text { with } V \in L^2_{\mathrm{uloc}} (\mathbb {R}) . \end{aligned}$$

*H* is a self-adjoint operator in $L^2(\mathbb {R})$, bounded from below. Assume that the spectrum of *H* has an isolated component $\sigma _0$, such that the range of the spectral projection $P_0$ corresponding to $\sigma _0$ has infinite dimension. Inspired by Kivelson [44], the authors of [71] consider the reduced position operator $P_0XP_0$ with domain $\mathcal {D}(P_0XP_0)=\mathcal {D}(X)\cap {{\,\mathrm{Ran}\,}}P_0$ and prove that:

(i) the resolvent of $P_0XP_0$ is a compact operator; hence, the spectrum of $P_0XP_0$ is purely discrete. The corresponding eigenvectors $\psi _{\gamma ,a} \in L^2(\mathbb {R})$ satisfy
  $$\begin{aligned} P_0XP_0 \psi _{\gamma ,a}=\gamma \psi _{\gamma ,a} \qquad \forall \,\gamma \in \sigma (P_0XP_0) ,\,1 \le a \le m(\gamma ), \end{aligned}$$
  where $m(\gamma )$ is the degeneracy index of the eigenvalue $\gamma$;
(ii) there exist two constants $0<\alpha , M <\infty$ such that
  $$\begin{aligned} \int _{\mathbb {R}} |\psi _{\gamma ,a}(\mathbf{x})|^2 \mathrm {e}^{ \alpha \Vert \mathbf{x}-\gamma \Vert } \, \mathrm{d}\mathbf{x}\le M \quad \forall \,\gamma \in \sigma (P_0XP_0) ,\,1 \le a \le m(\gamma ). \end{aligned}$$

Setting $\mathfrak {D}_0 = \sigma (P_0XP_0)$, the orthonormal basis $\left\{ \psi _{\gamma ,{a}}\right\} _{\gamma \in \mathfrak {D}_0, 1 \le a \le m(\gamma ) }$ is a GWB *in the sense of Nenciu–Nenciu*, as defined in [71], exponentially localized around the discrete set $\mathfrak {D}_0$. To prove that $\left\{ \psi _{\gamma ,{a}}\right\}$ is a GWB according to our (stronger) Definition 2.5, one has to show that $\exists \, m_* : m(\gamma )\le m_*$ for all $\gamma \in \mathfrak {D}$, which is not known in general. Whenever the Hamiltonian is periodic, the generalized Wannier basis constructed with the previous strategy [71] coincides with a usual composite Wannier basis [25, 64]. Furthermore, in [26] it has been proved that the number of eigenvalues of $P_0XP_0$, counted with their multiplicity, contained in an interval of length *L*, grows at most linearly with *L*. However, this result does not imply that the set $\sigma (P_0XP_0)$ is *r*-uniformly discrete or *R*-uniformly nowhere sparse for some $r,R>0$. The proof of the latter claims is, to our knowledge, still an open problem.

The previous construction for one-dimensional systems does not directly generalize to higher dimensions, essentially for two reasons:

(i) generically, the compact resolvent property of $P_0XP_0$ holds true only in $L^2(\mathbb {R})$, as one can easily see for $d=2$ when $P_0$ is $\mathbb {Z}^2$-periodic;
(ii) in general $P_0 X_i P_0$ and $P_0 X_jP_0$ do not commute for $i \ne j$, so the attempt to simultaneously diagonalize them fails. It is worth noticing that the topological and transport properties of the system are encoded exactly in the commutator between $P_0 X_1 P_0$ and $P_0 X_2 P_0$; see Sect. 2.2 and specifically equation (2.14). The relation with the theory of almost-commuting operators has been explored in [37].

Some proposals to circumvent these difficulties appeared recently in [79, 80], where the authors show that, under the additional crucial assumptions of uniform spectral gaps for the spectrum of the operator $PX_1P$, it is possible to prove the existence of an exponentially decaying GWB for the projection *P*. Proving that $PX_1P$ satisfies such uniform spectral gaps hypothesis is still an open problem, but in [79] the authors present numerical simulations showing that in explicit tight-binding models the spectrum of $PX_1P$ has spectral gaps only when the projection *P* is Chern trivial, which is in accordance with Conjecture 3.2.

Despite the difficulties to deal with *generic* non-periodic systems for $d>1$, there are several specific *d*-dimensional models for which the existence of a well-localized GWB has been proved, as in the following examples.

#### Example 2.8

*(d*-*dimensional insulators with weak disorder).* Generalized Wannier bases can be useful in the analysis of periodic systems perturbed by impurities and weak disorder. Given $\lambda \in \mathbb {R}$ and a Bravais lattice $\Gamma \subset \mathbb {R}^d$, $d\le 3$, we consider the Hamiltonian operator, acting in $L^2(\mathbb {R}^d)$,

$$\begin{aligned} H_{\lambda }=-\Delta + V_{\Gamma } + \lambda W \end{aligned}$$

where $V_\Gamma \in L^2_{\mathrm{uloc}}(\mathbb {R}^d)$ is $\Gamma$-periodic. Assume that $W\in L^2_{\mathrm{uloc}}(\mathbb {R}^d)$, so that $H_\lambda$ is an entire family of type *A* in the sense of Kato. The potential $V_\Gamma$ models the periodic background of the crystalline insulator, while *W* models either a sum of localized impurities or a delocalized disorder.

Assume that the spectrum of $H_0$ has an isolated component $\sigma _0$. Since $H_0$ is time-reversal symmetric, the projection $P_0$ onto the isolated component $\sigma _0$ admits a Wannier basis $\left\{ \psi _{\gamma ,a}:=\psi _{0,{a}}(\cdot -\gamma )\right\} _{\gamma \in \Gamma , 1\le a \le m}$, for some $m>0$, where each Wannier function $\psi _{\gamma ,{a}}$ is exponentially localized around $\gamma$ in the sense of Definition 2.5. By standard perturbation theory [41, Remark VII.2.3, p. 379] one can prove that there exists a $\lambda _0>0$ small enough such that, for $|\lambda |<\lambda _0$, the spectrum of $H_\lambda$ has an isolated component $\sigma _{\lambda }$ that varies continuously with $\lambda$ in the Hausdorff distance. Denote by $P_\lambda$ the spectral projection onto $\sigma _\lambda$.

In [70], it is shown how to transport an exponentially localized GWB from the range of $P_0$ to the range of $P_\lambda$. Let us review here the main steps of the proof rewritten in our setting. First of all, since $H_\lambda$ is an analytic family of type A, the family of projections is analytic in $\lambda$ [41, Theorem VII.3.1.7], in particular $\Vert P_\lambda -P_{\lambda '}\Vert \le C |\lambda -\lambda '|$ for some positive constant *C*. It follows that, for $|\lambda -\lambda '|$ small enough, there exists a unitary operator which intertwines the two projections and, as a consequence, it unitarily maps an orthonormal basis for the range of $P_\lambda$ into an orthonormal basis for the range of $P_{\lambda '}$. Furthermore, in view of the hypothesis on the Hamiltonian and the gap condition, one can prove that the projections, $P_\lambda$ and $P_{\lambda '}$, are both exponentially localized projection in the sense of Definition 2.3. In addition, using again the Riesz formula, together with the Combes–Thomas rotation and the relative smallness of *W*, one gets that, for some $\delta >0$ small enough, $\sup _{\mathbf{{a}} \in \mathbb {R}^d}\Vert {{\,\mathrm{e}\,}}^{-\delta \langle \cdot -\mathbf{{a}\rangle }} \left( P_{\lambda }-P_{\lambda '}\right) {{\,\mathrm{e}\,}}^{\delta \langle \cdot -\mathbf{{a}\rangle }}\Vert \le C |\lambda -\lambda '|$ which implies that the intertwining unitary, explicitly given by the Kato–Nagy formula, preserves the exponential localization; see [70]. Therefore, starting from an exponentially localized GWB for $P_0$ and iterating the unitary transport a finite number of times, one obtains a GWB for every $P_\lambda$, with $|\lambda |<\lambda _0$, which is exponentially localized around the same discrete lattice $\Gamma$ as the original one.

Notice that this argument relies on the fact that $P_\lambda$ is a spectral projection onto an isolated component of the spectrum, namely we are assuming that the Fermi energy lies always in a spectral gap. In the setting of random Schrödinger operator, it is usually considered also the case of the Fermi energy lying in a region of mobility gap. In such a situation, even though the integral kernel of the Fermi projection is not exponentially localized, it might still be possible to construct special orthonormal basis that is localized in space; see, for example, [32] and references therein.

#### Example 2.9

*(Deformed* *d*-*dimensional insulators: the Gubanov model).* A class of examples of non-periodic gapped quantum systems that admit an exponentially localized GWB is provided by a Schrödinger operator modeling the deformation of a periodic *d*-dimensional insulator. We recall the definition of this model, following [10], where it is called *quasicrystalline model of Gubanov*.

The Hamiltonian $H_0$ describing the undeformed system, acting in $L^2(\mathbb {R}^d)$ for $d \le 3$, is given by

$$\begin{aligned} H_0=-\Delta + V \qquad \text { with } V \in L^2_{\mathrm{uloc}}(\mathbb {R}^d). \end{aligned}$$

We assume that the spectrum of $H_0$ has an isolated component $\sigma _0$, and denote by $P_0$ the corresponding spectral projection. In order to describe the deformation of the system, we consider $g \in C^2(\mathbb {R}^d,\mathbb {R}^d)$ and $\Omega \subset \mathbb {R}^d$ and set

$$\begin{aligned} \begin{aligned}&b(g;\mathbf{x}) = \max _{1\le i,j,k\le d} \left[ |\partial _{i}\partial _{j}g_k(\mathbf{x})|\, , |\partial _{i}g_k(\mathbf{x})| \right] \\&b(g;\Omega )=\sup _{\mathbf{x}\in \Omega } b(g;\mathbf{x}) . \end{aligned} \end{aligned}$$

Let $V_g$ be the potential given by $V_g(\mathbf{x})=V(\mathbf{x}+g(\mathbf{x}))$, for all $\mathbf{x}\in \mathbb {R}^d$. Then the Hamiltonian of the deformed system is $H_g=-\Delta + V_g$.

Assume that $b(g,\mathbb {R}^d)=\xi < + \infty$ and that $P_0$ admits a GWB which is exponentially localized around some *r*-uniformly discrete set $\mathfrak {D}$. A consequence of [10, Proposition 4] is that, for $\xi$ small enough, the spectrum of $H_g$ has an isolated component $\sigma _{g}$ that varies continuously with $\xi$ in the Hausdorff distance. The corresponding spectral projection $P_g$ of $H_g$ is not norm continuous with respect to $\xi$. However, if, for $\xi$ small enough, one defines the unitary operator [10] $(Y\psi )(\mathbf{x})= \left( \det \left( |\partial _{j}(\mathbf{x}+g(\mathbf{x}))_i|\right) \right) ^{1/2} \psi (\mathbf{x}+g(\mathbf{x}))$ for every $\psi \in L^2(\mathbb {R}^2)$, then $YP_gY^*$ depends continuously on $\xi$; hence, $\left\| YP_gY^* - P_0 \right\| < 1$ for $\xi$ sufficiently small. Therefore, by repeating the steps described in Example 2.8 one can unitarily transport the GWB from the range of $P_0$ to the range of $YP_gY^*$, without spoiling the localization properties. Let $\{\psi _{\gamma ,a}\}_{\gamma \in \mathfrak {D}, 1 \le a \le m(\gamma )}$ be the GWB of $YP_gY^*$. Exploiting the explicit expression of the operator $Y^*$ and the fact that the derivatives of *g* are uniformly bounded by $\xi$, one gets that $\{\varphi _{{\widetilde{\gamma }},a}:=Y^* \psi _{\gamma ,a}\}_{{\widetilde{\gamma }} \in {\widetilde{\mathfrak {D}}}, 1 \le a \le m({\widetilde{\gamma }})}$ is an exponentially localized GWB for the range of $P_g$, where ${\widetilde{\mathfrak {D}}}:=\{\mathbf{x}\in \mathbb {R}^d \; |\; \mathbf{x}= \gamma +g(\gamma ), \gamma \in \mathfrak {D}\}$ is an $r'$-uniformly discrete set and $m({\widetilde{\gamma }})=m(\gamma )$.

#### Example 2.10

*(Magnetic perturbations of Chern-trivial 2D insulators).* Generalized Wannier functions naturally appear when a two-dimensional system is subjected to a constant magnetic field whose flux through the periodicity cell does not necessarily satisfies a commensurability condition with respect to the quantum of magnetic flux. While it is not possible, in such a case, to rely on any type of magnetic Bloch–Floquet transform, it is still possible to exploit the covariance with respect to magnetic translations. This problem has been tackled in [19] and [22]. Consider a $\mathbb {Z}^2$-periodic insulator modeled by a Bloch–Landau Hamiltonian, acting in $L^2(\mathbb {R}^2)$, that is

2.10 $$\begin{aligned} H_{\epsilon }=\left( -\mathrm {i}\nabla - \mathbf {A}_{\mathbb {Z}^2} -b_0\mathbf {A}-\epsilon \mathbf {A} \right) ^2 + V_{\mathbb {Z}^2} \end{aligned}$$

where $V_{\mathbb {Z}^2}$ is a smooth $\mathbb {Z}^2$-periodic scalar potential, $\mathbf {A}_{\mathbb {Z}^2}$ is a smooth $\mathbb {Z}^2$-periodic vector potential, $\epsilon \in \mathbb {R}$, $b_0 \in 2\pi \mathbb {Q}$ and $\mathbf {A}(\mathbf{x})=\frac{1}{2}\left( -x_2,x_1\right)$ is the magnetic potential of a constant magnetic field in the symmetric gauge. Assume that the spectrum of $H_0$ has an isolated component $\sigma _0$ and let $P_0$ be the corresponding spectral projection. Then, for $|\epsilon |$ small enough, the spectrum of $H_\epsilon$ has an isolated component $\sigma _{\epsilon }$ which varies continuously with $\epsilon$ in the Hausdorff distance. If $P_0$ has a vanishing Chern number, then $P_0$ admits a (magnetic) Wannier basis $\left\{ \psi _{\gamma ,{a}}\right\} _{\gamma \in \mathbb {Z}^2, 1\le a \le m}$, for some $m>0$, where each Wannier function $\psi _{\gamma ,{a}}$ is exponentially localized around $\gamma$. A consequence of the results in [19] is that, if $|\epsilon |$ is small enough, then $P_\epsilon$ admits a GWB exponentially localized around the same lattice $\mathbb {Z}^2$. Notice that the “magnetic” GWB for the perturbed projection $P_\epsilon$ present an *almost-ladder structure*, in the sense that it can be written just using a finite set of vectors together with magnetic translation and a position dependent phase, as explained in [22], where also a constructive algorithm for the GWB is provided.

### A Topological Marker in Position Space

The TKNN approach to the quantum Hall effect [82] established that the Hall conductivity, as given by the Kubo formula for linear response, is always an integer in units of $\frac{e^2}{h} = \frac{1}{2\pi }$. Shortly after, the topological origin of such an integer has been recognized [4, 78], thus establishing a *Transport–Topology Correspondence*: For gapped periodic magnetic systems, the Hall conductivity equals (up to a universal constant) the Chern number $c_1(P)$ of the (magnetic) Bloch bundle corresponding to the Fermi projection. Later, Haldane noticed that such a correspondence and the existence of a non-trivial topology only rely on the breaking of TRS, thus paving the way to the flourishing new field of topological insulators [35, 36].

The former Transport–Topology Correspondence for periodic systems has been later extended to non-periodic models, either by methods from non-commutative geometry [6, 7, 66], or by using the index of a pair of projections [5, 6]. As mentioned in the Introduction, in the non-periodic setting the Chern number is replaced by the ***Chern character*** *C*(*P*), given by formula (1.3) [6, 7]. Its main advantage is that it provides a topological marker defined *in position space*, with no reference to a quasi-momentum space which is available only in a periodic setting.

We emphasize that the Chern character can be defined without any ergodicity or covariance assumption on *P*, as in the following

#### Definition 2.11

*(Chern character).* Let *P* be an orthogonal projection in $L^2(\mathbb {R}^2)$. Let $X_1$ and $X_2$ be the multiplication operators by the respective component of the position operator, *i. e. *, $(X_i \psi )(\mathbf{x})=x_i \psi (\mathbf{x})$, for $\psi \in \mathcal {D}(X_i)$. Given $L>1$ and $\Lambda _L:= [-L,L]^2$, we denote by $\chi _{\Lambda _L}$ the characteristic function of the set $\Lambda _L$. Assume that $\left[ X_1, P \right] , \left[ X_2,P \right]$ uniquely extend to bounded operators and $\chi _{\Lambda _L} P \left[ \left[ X_1, P \right] ,\left[ X_2,P \right] \right] P\chi _{\Lambda _L}$ is a trace class operator for every $L>1$.

Under these assumptions, the *Chern character* of *P* is defined by the following trace per unit volume:

2.11 $$\begin{aligned} \begin{aligned} C(P)&:= \lim \limits _{L \rightarrow \infty } \frac{2\pi }{4L^2} {{\,\mathrm{Tr}\,}}\left( \mathrm {i}\chi _{\Lambda _L} P \Big [ \left[ X_1, P \right] ,\left[ X_2,P \right] \Big ] P \chi _{\Lambda _L}\right) \end{aligned} \end{aligned}$$

whenever the limit exists.

Formula (2.11) coincides up to a universal constant with the Hall conductivity in gapped systems, in the linear response regime, provided Kubo formula holds true. For the validity of the latter, see the recent papers [54, 56, 63, 81] and references therein; concerning the Kubo formula for *spin* conductivity, see [57, 58] and references therein.

The limit in (2.11) equals $2\pi$ times the trace per unit volume of the operator

2.12 $$\begin{aligned} \mathfrak {C}_{P}:= {\mathrm {i}} P \left[ \left[ X_1, P \right] ,\left[ X_2,P \right] \right] P, \end{aligned}$$

hence, it agrees with formula (1.3) in the Introduction. It is interesting to notice that it is possible to rewrite (2.12) in terms of commutators of the so-called *reduced position operators*. Let *P* be an exponentially localized projection and $\widetilde{X}_j\;:=\; P X_j P$ be the reduced position operator in direction $j \in \{1,2\}$. A direct computation^7^, exploiting only $P^2=P$ and $\left[ X_1,X_2\right] \;=\;0$, yields

2.13 $$\begin{aligned} \chi _{\Lambda _L} \mathfrak {C}_P=\chi _{\Lambda _L}\mathrm {i}P X_1 P X_2 P - \chi _{\Lambda _L} \mathrm {i}P X_2 P X_1 P \;=\; \chi _{\Lambda _L} \mathrm {i}\big [\widetilde{X}_1,\widetilde{X}_2\big ], \end{aligned}$$

so that

2.14 $$\begin{aligned} C(P) = 2\pi \, \tau \left( \mathrm {i}\big [\widetilde{X}_1,\widetilde{X}_2\big ] \right) , \end{aligned}$$

where $\tau (\cdot )$ denotes the trace per unit volume, which is *conditionally cyclic*^8^. Notice that the operator $\big [\widetilde{X}_1,\widetilde{X}_2\big ]$ is densely defined since the projection *P* maps pointwise exponentially decaying functions, with exponential decay less than $\beta$ in (2.2), into pointwise exponentially decaying functions.

Definition 2.11 contains two relevant constraints: the trace class condition of $\chi _{\Lambda _L}\mathfrak {C}_{P}\chi _{\Lambda _L}$ and the existence of the limit of (2.11), which are not trivial when *P* is a generic orthogonal projection. Whenever the orthogonal projection is exponentially localized and time-reversal symmetric, the Chern character vanishes, independently of any periodicity hypothesis, *cf. *[5, Theorem 3.9], as detailed in the following.

#### Proposition 2.12

(Chern character vanishes under TRS). Let *P* be an exponentially localized projection acting on $L^2(\mathbb {R}^2)$. Let time-reversal symmetry be encoded by^9^ an antiunitary operator $\Theta$ such that $\Theta ^2=\pm \mathbbm {1}$ and $[\Theta , X_i]=0$ for any $i\in \{1,2\}$. If *P* is time-reversal symmetric in the sense that $\Theta P \Theta ^{-1}= P$, then $C(P)=0$.

#### Proof

First, notice that by Proposition 4.1, the sequence of traces defining the Chern character is well defined. Then, by using that ${{\,\mathrm{Tr}\,}}(A)=\overline{{{\,\mathrm{Tr}\,}}(\Theta \,A\, \Theta ^{-1})}$ for every trace class operator *A* and exploiting the time-reversal symmetry of *P*, we have the chain of equalities

$$\begin{aligned}&\mathrm {i}{{\,\mathrm{Tr}\,}}\left( \chi _{\Lambda _L} P \left[ \left[ X_1, P \right] ,\left[ X_2,P \right] \right] P \chi _{\Lambda _L}\right) \\&\qquad =\mathrm {i}\, \overline{{{\,\mathrm{Tr}\,}}\left( \Theta \chi _{\Lambda _L} P \left[ \left[ X_1, P \right] ,\left[ X_2,P \right] \right] P \chi _{\Lambda _L}\Theta ^{-1}\right) }\\&\qquad =\,\mathrm {i}\, {{\,\mathrm{Tr}\,}}\left( {\left( \chi _{\Lambda _L} P \left[ \left[ X_1, P \right] ,\left[ X_2,P \right] \right] P \chi _{\Lambda _L}\right) }^*\right) \\&\qquad =-\mathrm {i}\, {{\,\mathrm{Tr}\,}}\left( \chi _{\Lambda _L} P \left[ \left[ X_1, P \right] ,\left[ X_2,P \right] \right] P \chi _{\Lambda _L}\right) \end{aligned}$$

which implies that the Chern character is zero. $\square$

Notice that the exponential localization hypothesis in Proposition 2.12 is only used to justify the formal computations with the double commutator formula; however, a sufficiently fast polynomial decay of the integral kernel would be sufficient to prove the statement.

Even though the limit (2.11) defining the Chern character might not exist for generic orthogonal projections, this is never the case when *P* is an exponentially localized projection. Moreover, in such situation, the Chern character is an integer. This fact has first been proved in [29, Appendix, Proposition 3 and Remark 3] in the discrete setting and using a different terminology^10^, covering also the more general case of a mobility gap. The proof in [29] is based on the index of pair of projections defined in [5, 6, 66], and here we adapt their argument to our setting, *i. e. *, exponentially localized projections on the continuum. We notice that the profound reason for the quantization of such index can be traced back to the Fedosov formula for the index of elliptic operators [30, 39]. Before formulating the results, let us briefly remind the definition of the index of pair of projections.

Consider the unitary multiplication operator *U*, defined by $(U\psi )(\mathbf{x}) =U(\mathbf{x}) \psi (\mathbf{x})$ with

2.15 $$\begin{aligned} U(\mathbf{x}):= \left\{ \begin{aligned}&\frac{x_1+\mathrm {i}x_2}{\sqrt{x_1^2+x_2^2}}&\qquad x_1 + \mathrm {i}x_2 \notin [0,+\infty ) \\&1&\qquad \, x_1 + \mathrm {i}x_2 \in [0,+\infty ). \end{aligned}\right. \end{aligned}$$

The unitary *U* represents a singular gauge transformation associated with the insertion of a magnetic flux tube carrying one unit of quantum flux. As emphasized in [5], it is not restrictive to consider only unitary operators with unit winding number, like (2.15), see [5, Theorem 4.2]. Then, the index of pair of projections *P*, $UPU^*$, is defined by

2.16 $$\begin{aligned} {\text {Index}}(P,UPU^*):= & {} \dim \left( \ker (P-UPU^*-1)\right) \nonumber \\&-\dim \left( \ker (UPU^*-P-1)\right) \in \mathbb {Z}. \end{aligned}$$

We show that the Chern character coincides with the index of a pair of projection and hence is an integer, with no ergodicity or covariance assumption on *P*.

#### Proposition 2.13

Let *P* be an orthogonal projection acting on $L^2(\mathbb {R}^2)$ which is an exponentially localized projection in the sense of Definition 2.3. Let *U* be the unitary multiplication operator defined in (2.15). Then,

2.17 $$\begin{aligned} C(P)={\text {Index}}(P,UPU^*) \end{aligned}$$

where *C*(*P*) is the Chern character defined in (2.11) and ${\text {Index}}(P,UPU^*)$ is the index of pair of projections defined in (2.16).

The proof is postponed to Appendix A.

## Main Result

We can now formulate our main result.

### Theorem 3.1

(Localization of GWB implies Chern triviality). Let *P* be an orthogonal projection acting on $L^2(\mathbb {R}^2)$, which is an exponentially localized projection in the sense of Definition 2.3. Let $\mathfrak {D}\subset \mathbb {R}^2$ be a *r*-uniformly discrete set and let $s>4$. Suppose that *P* admits a generalized Wannier basis $\{\psi _{\gamma ,a}\}_{\gamma \in \mathfrak {D}, 1 \le a \le m(\gamma )}$ which is *s*-localized around $\mathfrak {D}$, in the sense of Definition 2.5. Then, the Chern character of *P* is zero.

Notice that periodicity plays no role in Theorem 3.1. In non-periodic context, one might think that a natural generalization of Bravais lattice is a *Delone set*, which is both *r*-uniformly discrete and *R*-uniformly nowhere sparse$^(5)$. However, in the proof of the above theorem only the property of ${\mathfrak {D}}$ to be uniformly discrete is used, via the“Generalized Maclaurin–Cauchy test” (Lemma B.1).

As we have already mentioned in the Introduction, in the periodic context the relation between the localization of Wannier bases and the topology of the Bloch bundle is a well-established paradigm [62]. Inspired by the latter result, we conjecture that the remarkable dichotomic character that pertains to periodic systems is actually a more general phenomenon.

### Conjecture 3.2

(Localization dichotomy for non-periodic gapped systems). Let $P_0$ be the spectral projection, acting on $L^2(\mathbb {R}^2)$, corresponding to an isolated component $\sigma _0$ of an operator satisfying the hypotheses of Proposition 2.4. Then there exists a number $s_* \ge 1$ such that the following statements are equivalent:

- $P_0$ admits a generalized Wannier basis that is exponentially localized around a *r*-uniformly discrete set, for some $r >0$.
- $P_0$ admits a generalized Wannier basis that is *s*-localized, with $s \ge s_*$, around a $r'$-uniformly discrete set, for some $r' >0$.
- $P_0$ is Chern trivial in the sense that its Chern character $C(P_0)$ exists and is equal to zero.

Clearly, (a) implies (b) by a simple inequality. The implication from (b) (with $s_*>4$) to (c) is the content of Theorem 3.1, which is proved in the next section. The open problem in the conjecture is to show that (c) is implies (a). Moreover, since for any time-reversal symmetric system the Chern character vanishes, see Proposition 2.12, proving that (c) is implies (a) would solve a long standing conjecture about the existence of generalized Wannier bases for time-reversal symmetric systems [70].

Furthermore, in view of the periodic counterpart, the threshold $s_*>4$ appearing in Theorem 3.1 does not seem optimal. In fact, a generalization of the localization dichotomy for periodic systems proved in [62] would require $s_*=1$.

Theorem 3.1, coupled with the techniques used in [70], allows to show that the nonexistence of a well-localized GWB is stable with respect to suitable perturbations, as detailed in the following Corollary. A similar stability result concerning the vanishing of the Chern character holds also true.

### Corollary 3.3

(Stability of the GWB delocalization). Consider the family of Hamiltonian operators, acting in $L^2(\mathbb {R}^2)$,

$$\begin{aligned} H_\lambda =\frac{1}{2}(-\mathrm {i}\nabla - b_0\mathbf{{A}}-\mathbf {A}_{\mathbb {Z}^2})^2 + V_{\mathbb {Z}^2}+ \lambda W \, , \qquad \lambda \in \mathbb {R}\end{aligned}$$

where $V_{\mathbb {Z}^2}$ is a smooth $\mathbb {Z}^2$-periodic scalar potential, $\mathbf {A}_{\mathbb {Z}^2}$ is a smooth $\mathbb {Z}^2$-periodic vector potential, $b_0 \in 2\pi \mathbb {Q}$ and ${\mathbf {A}}(\mathbf{x})=\frac{1}{2}\left( -x_2,x_1\right)$. Assume that *W* is infinitesimally relatively bounded with respect to $H_0$ and that the spectrum of $H_0$ has an isolated component $\sigma _0\subset (E_-,E_+)$, $E_\pm \in \mathbb {R}\setminus \sigma (H_0)$. For $|\lambda |$ small enough, say $|\lambda | < \delta$, $E_\pm$ belong to the resolvent set of $H_{\lambda }$. Denote by $P_\lambda$ the spectral projection onto the spectral island $\sigma _\lambda :=\sigma (H_\lambda ) \cap (E_-,E_+)$. It holds true that

(i) if the Chern number of $P_0$ is different from zero, then for $|\lambda | < \delta$ the projection $P_\lambda$ does not admit any GWB that is *s*-localized for $s>4$;
(ii) if $P_0$ admits an *s*-localized GWB around a certain *r*-uniformly discrete set for $s>4$, then $C(P_\lambda )=0$ for $|\lambda |<\delta$.

### Proof

Let us prove statement (i). We first consider $\lambda =0$. Suppose by contradiction that $P_0$ admits a *s*-localized GWB, with $s>4$. Then, in view of Theorem 3.1, the Chern character of $P_0$ vanishes. This leads to a contradiction since, as $P_0$ commutes with a unitary representation of $\mathbb {Z}^2$, one has $C(P_0) = c_1(P_0) \ne 0$. Consider now $\lambda \ne 0$. Since *W* is infinitesimally small with respect to $H_0$, $H_\lambda$ defines an entire family of type A; hence, for $|\lambda |$ small enough $E_\pm$ belongs to the resolvent set of $H_\lambda$ and $\sigma _\lambda$ is a well-defined isolated component of the spectrum [41, Remark VII.2.3, p. 379], such that $\Vert P_\lambda - P\Vert \le C |\lambda |<1$. Assume now by contradiction that $P_\lambda$ admits a GWB that is *s*-localized for $s>4$. Therefore, following the idea in [70], it is possible to unitarily transport the GWB back to the original system by using the Kato–Nagy unitary operator *U* that intertwines $P_0$ and $P_\lambda$. Moreover, since $H_\lambda$ satisfies the assumptions of Proposition 2.4, we can apply the result of [22, Lemma C.1], which implies that $U-\mathbbm {1}$ is an integral operator with an exponentially localized kernel, that is a kernel satisfying (2.2). Hence, via a Schur–Hölmgren estimate we get that

$$\begin{aligned} \sup _{\mathbf{{a}} \in \mathbb {R}^2} \Vert \langle \cdot - \mathbf{{a}} \rangle ^{s} U \langle \cdot - \mathbf{{a}} \rangle ^{-s} \Vert < +\infty \end{aligned}$$

which implies that *U* preserves the localization properties of the GWB. Therefore, $P_0$ admits a GWB that is *s*-localized. Then, Theorem 3.1 implies that the $C(P_0)=0$, which is a contradiction.

The proof of statement (ii) follows a similar argument. $\square$

## Proofs

### Well-Posedness of the Chern Character

The main result of this section is the proof that the operator $\chi _{\Lambda _L} \mathfrak {C}_P \chi _{\Lambda _L}$ with *P* an exponentially localized projection, see (2.12), is trace class, and that its trace is ${\mathcal {O}}(L^2)$.

As anticipated in Sect. 2.2, the topological properties of the system are encoded in the commutator $\left[ \widetilde{X}_1,\widetilde{X}_2\right]$, and the Chern character is proportional to

4.1 $$\begin{aligned} \tau \left( \mathfrak {C}_P \right) =\tau \left( \mathrm {i}\left[ \widetilde{X}_1,\widetilde{X}_2\right] \right) \;=\; \lim \limits _{L \rightarrow + \infty } \frac{1}{4 L^2} {{\,\mathrm{Tr}\,}}\left( \chi _{\Lambda _L}\, \mathrm {i}\,\left[ \widetilde{X}_1,\widetilde{X}_2\right] \chi _{\Lambda _L}\right) . \end{aligned}$$

Now, we prove that for all $L>1$, each summand of the commutator $\chi _{\Lambda _L} [\widetilde{X}_1,\widetilde{X}_2]\chi _{\Lambda _L}$, and the terms involved in the direct computation (2.13) are trace class.

#### Proposition 4.1

Let *P* be an exponentially localized projection acting on $L^2(\mathbb {R}^2)$, let $X_i$, for $i \in \left\{ 1,2 \right\}$, denote the components of the position operator, and let $\chi _{\Lambda _L}$ be the characteristic function of the set $\Lambda _L:=[-L,L]^2$. Then

(i) for every $m,n,m',n' \in \mathbb {N}= \left\{ 0,1,\ldots \right\}$ the operator
  $$\begin{aligned} \chi _{\Lambda _L} P (X_i)^m(X_j)^{m'} P (X_j)^n (X_i)^{n'} \end{aligned}$$
  is a trace class operator.
(ii) Moreover, there exists $C>0$ such that
  $$\begin{aligned} \left| {{\,\mathrm{Tr}\,}}(\chi _{\Lambda _L} P \left[ \left[ X_1, P\right] ,\left[ X_2, P\right] \right] \chi _{\Lambda _L})\right| \le C L^2 \qquad \forall L \ge 1. \end{aligned}$$

#### Proof

(i). Consider $\beta$ as in inequality (2.2). Let $\eta <\beta /3$ and consider the multiplication operators ${\mathrm {e}}^{-\eta \Vert X \Vert }$ and ${\mathrm {e}}^{\eta \Vert X \Vert }$. One has

$$\begin{aligned}&\chi _{\Lambda _L} P (X_i)^m (X_j)^{m'}P (X_j)^n(X_i)^{n'}\\&\qquad = \chi _{\Lambda _L} \, {\mathrm {e}}^{4\eta \Vert X\Vert }\, {\mathrm {e}}^{-4\eta \Vert X \Vert } \, P \, {\mathrm {e}}^{2\eta \Vert X\Vert } \, (X_i)^m (X_j)^{m'} \, {\mathrm {e}}^{-2\eta \Vert X \Vert }\, P \, (X_j)^n (X_i)^{n'} \, . \end{aligned}$$

From estimate (2.2) and the triangle inequality it follows that

$$\begin{aligned}&\left| \left( {\mathrm {e}}^{-4\eta \Vert X \Vert } \, P \, {\mathrm {e}}^{2\eta \Vert X\Vert } \, (X_i)^m (X_j)^{m'} \right) (\mathbf{x},\mathbf{y}) \right| \le C {\mathrm {e}}^{-\eta \Vert \mathbf{x}\Vert } {\mathrm {e}}^{-(\beta -3\eta ) \Vert \mathbf{x}-\mathbf{y}\Vert } \, ,\\&\qquad \left| \left( {\mathrm {e}}^{-2\eta \Vert X \Vert }\, P \, (X_j)^n (X_i)^{n'} \right) (\mathbf{x},\mathbf{y}) \right| \le C' {\mathrm {e}}^{-(\beta -\eta ) \Vert \mathbf{x}-\mathbf{y}\Vert } {\mathrm {e}}^{-\eta \Vert \mathbf{x}\Vert }\, . \end{aligned}$$

Thus, the two operators appearing on the l.h.s above have both integral kernels in $L^2(\mathbb {R}^2 \times \mathbb {R}^2)$ and hence are Hilbert–Schmidt operators. Since the product of two Hilbert–Schmidt operators is in the trace class ideal, and $\chi _{\Lambda _L} \, {\mathrm {e}}^{4\eta \Vert X \Vert }$ is a bounded operator, the first part of the proposition is proved. In particular, choosing $m=0=m'$ and $n=0=n'$, one concludes that $\chi _{\Lambda _L}P^2=\chi _{\Lambda _L}P$ is trace class.

(ii). We have that

$$\begin{aligned} \begin{aligned} \left| {{\,\mathrm{Tr}\,}}(\chi _{\Lambda _L} \mathfrak {C}_P \chi _{\Lambda _L})\right|&= \left| {{\,\mathrm{Tr}\,}}(\chi _{\Lambda _L} \mathfrak {C}_P P \chi _{\Lambda _L})\right| \le \Vert \chi _{\Lambda _L} \mathfrak {C}_{P} P \chi _{\Lambda _L} \Vert _1 \\&\le \Vert \chi _{\Lambda _L} \mathfrak {C}_{P} P \Vert _2 \Vert P \chi _{\Lambda _L} \Vert _2 . \end{aligned} \end{aligned}$$

By writing explicitly the integral kernel of $\mathfrak {C}_{P}$ and exploiting that the kernel of *P* is jointly continuous and satisfies (2.2), we obtain that there exist two positive constants $\alpha$ and *C* such that

$$\begin{aligned} |\left( \chi _{\Lambda _L} \mathfrak {C}_{P} P \right) (\mathbf{x},\mathbf{y})| \le C \chi _L(\mathbf{x}) {\mathrm {e}}^{-\alpha \Vert \mathbf{x}-\mathbf{y}\Vert } . \end{aligned}$$

Hence, we can easily estimate the Hilbert–Schmidt norm of $\chi _{\Lambda _L} \mathfrak {C}_{P} P$ and $P \chi _{\Lambda _L}$ by explicit integration and conclude the proof of the proposition. $\square$

### The Proof in a Nutshell

Let *P* be an exponentially localized projection that admits a GWB localized around a *r*-uniformly discrete set $\mathfrak {D}$, with localization function *G* (see Definition 2.5). In general, one cannot expect the operators $\widetilde{X}_i$ to be diagonal in the GWB representation, as the expectation value of the position operators in the GWB, namely $\langle \psi _{\gamma ,{a}}, X_i \psi _{\eta ,{b}} \rangle \, ,$ has nonvanishing off-diagonal elements. However, in order to understand the main idea behind our proof, let us suppose for the moment that *P* admits a GWB made of generalized Wannier functions localized on a *r*-uniformly discrete set $\mathfrak {D}$ and with compact and mutually disjoint supports. In this setting, we have

4.2 $$\begin{aligned} \langle \psi _{\gamma ,a}, X_i \psi _{\eta ,b} \rangle = \langle \psi _{\gamma ,a}, \widetilde{X}_i \psi _{\eta ,b} \rangle =: \delta _{\gamma ,\eta }\delta _{a,b}f_i(\gamma ,a), \qquad i\in \{1,2\} \,, \end{aligned}$$

in view of the mutually disjoint support property of the GWFs. Then, the operators $\widetilde{X}_1$ and $\widetilde{X}_2$ are diagonal operators in the generalized Wannier basis, which implies that $[\widetilde{X}_1,\widetilde{X}_2]=0$. Therefore, we can easily see from (4.1) that in such oversimplified setting the Chern character vanishes and Theorem 3.1 holds true.

Hence, we find convenient to introduce the operators $\Gamma _i$, for $i\in \left\{ 1,2\right\}$, by setting

4.3 $$\begin{aligned} \Gamma _i:= \sum _{\gamma \in \mathfrak {D}} \sum _{1\le a \le m(\gamma )} \gamma _i \left| \psi _{\gamma ,a} \right\rangle \left\langle \psi _{\gamma ,a} \right| \, , \end{aligned}$$

acting on the maximal domain $\mathcal {D}(\Gamma _i):= \left\{ \varphi \in L^2(\mathbb {R}^2) \; | \; \sum _{\gamma ,a} |\gamma _i|^2 |\langle \psi _{\gamma ,a}, \varphi \rangle |^2\right. \left. < +\infty \right\}$.

The orthonormality of the GWB implies that

$$\begin{aligned} \Gamma _i P = \Gamma _i = P \Gamma _i \, , \qquad \Gamma _i \Gamma _j = \Gamma _j \Gamma _i \, , \end{aligned}$$

and, obviously, one has $\tau ( \left[ \Gamma _i,\Gamma _j\right] ) = 0$. The strategy of the proof of Theorem 3.1 is to control “how far” the commutator between the reduced position operators is from the commutator between the operators $\Gamma _i$, when they are localized on the compact region $\Lambda _L$. In particular, we show that

4.4 $$\begin{aligned} \left| {{\,\mathrm{Tr}\,}}\left( \chi _{{\Lambda _L}} \left( \left[ \widetilde{X}_1,\widetilde{X}_2\right] -\left[ \Gamma _i,\Gamma _j\right] \right) \right) \right| = \mathcal {O}(L) , \end{aligned}$$

which implies that

$$\begin{aligned} \tau \left( \left[ \widetilde{X}_1,\widetilde{X}_2\right] \right) =\tau \left( \left[ \widetilde{X}_1,\widetilde{X}_2\right] -\left[ \Gamma _i,\Gamma _j\right] \right) =0. \end{aligned}$$

The proof consists in splitting the contribution of the difference of commutators appearing in (4.4) in several terms and to show that each of these terms separately goes at most linearly in *L* as $L \rightarrow \infty$. The main ingredients of our analysis are the estimates contained in the Proposition 4.2 which are of two types:

- Estimates (4.6) and (4.5) show that the $L^2$ norm contribution in $\Lambda _L$ (resp. in $\Lambda ^c_L$ ) coming from the GWFs that have a center outside $\Lambda _L$ (resp. inside $\Lambda _L$) is at most of order *L*. Loosely speaking, when we look at the norm of GWFs in a certain region of the plane, the error we make by considering only the ones with the center in such region grows only like the boundary of the region.
- The estimates (4.8-4.11) are used to analyze the contribution to the trace in (4.4) coming from the fact that the reduced position operators $\widetilde{X}_i$ are not diagonal in the generalized Wannier basis, namely (4.2) does not hold true in general. From the proof of (4.11), we have that such error is again of order *L* as $L\rightarrow \infty$.

### Proof of Theorem 3.1

To make the proof as clear as possible, we first recollect in the next technical proposition all the important estimates on the GWB that we need for the proof. Notice that in order to estimate the values of certain series over the *r*-uniformly discrete set $\mathfrak {D}$ we make use of a generalized Cauchy–Maclaurin test, see Lemma B.1, to obtain an upper bound using Lebesgue integrals over the plane.

#### Proposition 4.2

Let the hypotheses of Theorem 3.1 be satisfied. Then, there exist two positive constants $\mathcal {I}_{\textsc {mo}},\mathcal {I}_{\textsc {mi}}$ such that, for every $L\ge 1$, one has

4.5 $$\begin{aligned}&\sum \limits _{\xi \in \Lambda _L , \, c} \left\| \chi _{\Lambda _L^c} \psi _{\xi ,c}\right\| \le \mathcal {I}_{\textsc {mo}}\, L \, , \end{aligned}$$

4.6 $$\begin{aligned}&\sum \limits _{\xi \notin \Lambda _L \, , c} \left\| \chi _{\Lambda _L} \psi _{\xi ,c}\right\| \le \mathcal {I}_{\textsc {mi}}\, L \, . \end{aligned}$$

Moreover, there exists a function $F: [0,+ \infty ) \rightarrow [0,+ \infty )$ such that

4.7 $$\begin{aligned} \sum \limits _{a=1}^{m(\gamma )}\sum \limits _{b=1}^{m(\eta )}\, \left| \left\langle \psi _{\gamma ,a}, (X_i-\gamma _i) \psi _{\eta ,b} \right\rangle \right| \le F(\Vert \gamma -\eta \Vert ) \qquad \forall \, i\in \{1,2\} \, , \end{aligned}$$

and the following integrability conditions are satisfied:

4.8 $$\begin{aligned}&\int _{\mathbb {R}^2}\mathrm{d}\mathbf{x}\, F(\Vert \mathbf{x}\Vert ) =: I_1 < \infty \, , \end{aligned}$$

4.9 $$\begin{aligned}&\int _{\mathbb {R}^2}\mathrm{d}\mathbf{x}\, F(\Vert \mathbf{x}\Vert )^2 =: I_2 < \infty \,, \end{aligned}$$

4.10 $$\begin{aligned}&\int _{\mathbb {R}^2}\mathrm{d}\mathbf{x}\, F(\Vert \mathbf{x}\Vert )\,\Vert \mathbf{x}\Vert =: I_3 < \infty \, . \end{aligned}$$

4.11 $$\begin{aligned}&\lim \limits _{L \rightarrow + \infty } \frac{1}{L^2} \int _{\Lambda _L} \mathrm{d} \mathbf{x}\, \int _{\mathbb {R}^2 \setminus \Lambda _L} \mathrm{d} \mathbf{y}\, F^2(\Vert \mathbf{x}-\mathbf{y}\Vert ) \, =\, 0 \, . \end{aligned}$$

The proof of Proposition 4.2, postponed to Appendix B.3, shows that one can choose *F* in the form $F(\Vert \mathbf{x}\Vert ):= k_s \langle \mathbf{x}\rangle ^{-(s-2-\epsilon )}$, for an arbitrary $0< \epsilon <1$. However, we prefer to state the Proposition in the form above, to single out the properties of *F* that will be used in the proof of Theorem 3.1. Indeed, we now prove the statement of Theorem 3.1 using only the estimates (4.5)–(4.11). For the sake of better readability, in the following the generic series $\sum _{\gamma \in \mathfrak {D}\cap \Lambda _L} \sum _{1\le a \le m(\gamma )} A(\gamma ,a)$ and $\sum _{\gamma \in \mathfrak {D}} \sum _{1\le a \le m(\gamma )} A(\gamma ,a)$ will be written shortly as $\sum _{\gamma \in \Lambda _L ,a}A(\gamma ,a)$ and $\sum _{\gamma ,a}A(\gamma ,a)$, respectively.

Let us start by noticing that by a simple algebraic manipulation one has

$$\begin{aligned} \chi _{\Lambda _L}\left( \left[ \widetilde{X}_1,\widetilde{X}_2\right] - \left[ \Gamma _1,\Gamma _2\right] \right)&= \chi _{\Lambda _L}\left[ \left( \widetilde{X}_1-\Gamma _1\right) , \left( \widetilde{X}_2-\Gamma _2\right) \right] \\&\quad + \chi _{\Lambda _L} \left[ \left( \widetilde{X}_1-\Gamma _1\right) , \Gamma _2 \right] + \chi _{\Lambda _L} \left[ \Gamma _1, \left( \widetilde{X}_2-\Gamma _2\right) \right] \\&=:\, T_1 + T_2 + T_3 \, . \end{aligned}$$

In the following, we show that each of the traces ${{\,\mathrm{Tr}\,}}(T_i)$, $i \in \{1,2,3\}$, goes at most linearly in *L* as $L \rightarrow \infty$.

First of all, note that all the terms in the commutators appearing in $\chi _{\Lambda _L} T_j$, $j \in \{1,2,3\}$ are trace class due to Proposition B.4. Let us start by analyzing the trace of $\chi _{\Lambda _L} T_2$. By substituting $\chi _{\Lambda _L} = 1 - \chi _{{\Lambda ^c_L}}$ and by exploiting the fact that *P* is an orthogonal projection and that the GWB is an orthonormal basis of ${{\,\mathrm{Ran}\,}}(P)$, we obtain

$$\begin{aligned}&\left| \sum \limits _{\xi ,c}\sum \limits _{\gamma ,a} \left\langle \psi _{\xi ,c}, \chi _{\Lambda _L}\psi _{\gamma ,a} \right\rangle \left\langle \psi _{\gamma ,a}, (X_1-\gamma _1) \psi _{\xi ,c} \right\rangle \left( \xi _2- \gamma _2\right) \right| \\&\quad \le \sum \limits _{\xi \in \Lambda _L , \, c} \sum \limits _{\gamma ,a} \delta _{\gamma ,\xi } \delta _{a,c} | \left\langle \psi _{\gamma ,a}, (X_1-\gamma _1) \psi _{\xi ,c} \right\rangle \left( \xi _2- \gamma _2\right) | \\&\qquad + \sum \limits _{\xi \in \Lambda _L , \, c} \sum \limits _{\gamma ,a} \left| \left\langle \chi _{\Lambda _L^c}\psi _{\xi ,c}, \psi _{\gamma ,a} \right\rangle \left\langle \psi _{\gamma ,a}, (X_1-\gamma _1) \psi _{\xi ,c} \right\rangle \left( \xi _2- \gamma _2\right) \right| \\&\qquad + \sum \limits _{\xi \notin \Lambda _L \, , c} \sum \limits _{\gamma ,a} \left| \left\langle \chi _{\Lambda _L}\psi _{\xi ,c}, \psi _{\gamma ,a} \right\rangle \left\langle \psi _{\gamma ,a}, (X_1-\gamma _1) \psi _{\xi ,c} \right\rangle \left( \xi _2- \gamma _2\right) \right| \\&\quad =:T_{21}+T_{22}+T_{23} \, . \end{aligned}$$

The first series, namely $T_{21}$, is zero after the summation in $\gamma$. The series $T_{22}$ reads

4.12 $$\begin{aligned} \begin{aligned}&\sum \limits _{\xi \in \Lambda _L , \, c} \sum \limits _{\gamma ,a} \left| \left\langle \chi _{\Lambda _L^c}\psi _{\xi ,c}, \psi _{\gamma ,a} \right\rangle \left\langle \psi _{\gamma ,a}, (X_1-\gamma _1) \psi _{\xi ,c} \right\rangle \left( \xi _2- \gamma _2\right) \right| \\&\quad \quad \le \sum \limits _{\xi \in \Lambda _L , \, c} \Vert \chi _{\Lambda _L^c}\psi _{\xi ,c} \Vert \sum \limits _{\gamma \in \mathfrak {D}} F(\Vert \gamma -\xi \Vert ) \Vert \gamma -\xi \Vert \le K_r I_3 \mathcal {I}_{\textsc {mo}}\,\, L \, , \end{aligned} \end{aligned}$$

where we have used (4.5), (4.7) and (4.7), together with Lemma B.1 to estimate the series with the integral. Analogously, by using (4.7), Lemma B.1, (4.6) and (4.10), we get that $\left| T_{23}\right| \le K_r I_3 \mathcal {I}_{\textsc {mi}}\,\, L$.

Therefore, we have obtained an upper bound for the trace of $\chi _{\Lambda _L}T_2$, that is $\left| {{\,\mathrm{Tr}\,}}(\chi _{\Lambda _L} T_2)\right| \le C L$ as $L \rightarrow \infty .$ An upper bound on the trace of $\chi _{\Lambda _L} T_3$ is obtained by a similar computation: it goes also at most linearly in *L* as $L \rightarrow \infty$. Hence, both these terms do not contribute to the thermodynamic limit (4.1).

It remains to estimate the trace of $\chi _{\Lambda _L}T_1$. By similar computations as the ones above, we can write $\chi _{\Lambda _L}T_1=:R_1+R_2+R_3$, where $R_i$, $i\in \left\{ 1,2,3\right\}$ are (a posteriori absolutely convergent) series such that $R_3$ contains the localization function $\chi _{\Lambda _L^c}$, $R_2$ contains the localization function $\chi _{\Lambda _L}$ and $R_1$ does not contain neither $\chi _{\Lambda _L}$ nor $\chi _{\Lambda _L^c}$. By using (4.6), (4.5) and (4.8) in a similar way as in (4.12), one can show that $R_2$ and $R_3$ are absolutely convergent series that go at most linearly in *L*, as $L \rightarrow \infty$. This means that the only contribution to the limit $L\rightarrow \infty$ can come from $R_1$. However, this is not the case as we now show. Consider the series $R_1$:

$$\begin{aligned} \begin{aligned} R_1:=&\sum \limits _{\gamma ,a} \sum \limits _{\eta ,b} \sum \limits _{\xi \in \Lambda _L , \, c} \delta _{\gamma ,\xi } \delta _{a,c} \left\langle \psi _{\gamma ,a}, (X_1-\gamma _1) \psi _{\eta ,b} \right\rangle \left\langle \psi _{\eta ,b}, (X_2-\eta _2) \psi _{\xi ,c} \right\rangle \\&- \sum \limits _{\gamma ,a} \sum \limits _{\eta ,b} \sum \limits _{\xi \in \Lambda _L , \, c}\delta _{\gamma ,\xi } \delta _{a,c} \left\langle \psi _{\gamma ,a}, (X_2-\gamma _2) \psi _{\eta ,b} \right\rangle \left\langle \psi _{\eta ,b}, (X_1-\eta _1) \psi _{\xi ,c} \right\rangle \, . \end{aligned} \end{aligned}$$

By using Lemma B.1 and (4.9), one easily gets that $R_1$ is absolutely convergent together with a non-optimal estimate (quadratic in *L*) for its sum. Let us now show that also $R_1$ goes at most linearly in *L*. By using the shorthand notation $D(\eta ,\xi ,b,c):=\left\langle \psi _{\xi ,c}, (X_1-\xi _1) \psi _{\eta ,b} \right\rangle \left\langle \psi _{\eta ,b}, (X_2-\eta _2) \psi _{\xi ,c} \right\rangle$, $R_1$ can be written as

$$\begin{aligned} R_1&=\sum \limits _{\eta ,b} \sum \limits _{\xi \in \Lambda _L , \, c} D(\eta ,\xi ,b,c) - \sum \limits _{\eta \in \Lambda _L,b} \sum \limits _{\xi ,c} D(\eta ,\xi ,b,c) \\&= \sum \limits _{\eta \in \mathfrak {D}\setminus \Lambda _L, \, b}\,\, \sum \limits _{\xi \in \Lambda _L , \, c} D(\eta ,\xi ,b,c) - \sum \limits _{\eta \in \Lambda _L,b} \,\,\sum \limits _{\xi \in \mathfrak {D}\setminus \Lambda _L, \, c} D(\eta ,\xi ,b,c) \, . \end{aligned}$$

Notice that $|D(\eta ,\xi ,b,c)| \le F^2(\Vert \eta -\xi \Vert )\,$. For our purposes, we just need to study the asymptotics of the absolute value of the series of $R_1$, namely

$$\begin{aligned} |R_1|&\le \sum \limits _{\eta \in \mathfrak {D}\setminus \Lambda _L, \; b}\,\, \sum \limits _{\xi \in \Lambda _L , \, c}\left| D(\eta ,\xi ,b,c)\right| + \sum \limits _{\eta \in \Lambda _L,b}\,\, \sum \limits _{\xi \in \mathfrak {D}\setminus \Lambda _L, \; c} \left| D(\eta ,\xi ,b,c)\right| \\&\le 2 \sum \limits _{\eta \in \mathfrak {D}\setminus \Lambda _L}\,\, \sum \limits _{\xi \in \Lambda _L }F^2(\Vert \eta -\xi \Vert ). \end{aligned}$$

From the proof of Proposition 4.2, it is clear that *F* is not in $\ell ^1(\mathfrak {D}\times \mathfrak {D})$. As we cannot invoke Lebesgue dominated convergence theorem in order to perform the limit $L \rightarrow \infty$, we explicitly estimate the series with Lemma B.1, obtaining that

4.13 $$\begin{aligned} \lim _{L \rightarrow \infty } \frac{1}{4 L^2}\sum \limits _{\eta \in \mathfrak {D}\setminus \Lambda _L}\,\, \sum \limits _{\xi \in \Lambda _L } F^2(\Vert \eta -\xi \Vert )\le & {} K^2_r \lim \limits _{L \rightarrow + \infty } \frac{1}{ 4 L^2} \int _{\Lambda _L} \mathrm {d} \mathbf {x}\, \nonumber \\&\cdot \int _{\mathbb {R}^2 \setminus \Lambda _L} \mathrm {d} \mathbf {y}\, F^2(\Vert \mathbf {x}-\mathbf {y}\Vert ) \end{aligned}$$

where the limit vanishes in view of (4.11). Taking into account the last estimate, and all the previous ones, we get (4.4), and the proof is concluded.

## Appendix A. Proof of Proposition 2.13

Before starting with the proof of Proposition 2.13, let us recall some known facts about the index of a pair of projections. First, let $\mathbf{p} \in \mathbb {R}^2$ and $U_\mathbf{p}$ be the multiplication operator associated with the function $U(\cdot - \mathbf{p})$, clearly $U_0 \equiv U$. As it is proved in [5], we have that, for every $\mathbf{p} \in \mathbb {R}$

A.1 $$\begin{aligned} \begin{aligned}&{\text {Index}}(P,U_\mathbf {p}PU^*_\mathbf {p})={\text{ Index }}(P,UPU^*)={{\,\mathrm {Tr}\,}}((P-UPU^*)^3)\\&\qquad =\int _{\mathbb {R}^{6}} \mathrm {d} \mathbf {x}\mathrm {d} \mathbf {y}\mathrm {d} \mathbf {z}P(\mathbf {x}, \mathbf {y}) P(\mathbf {y}, \mathbf {z}) P(\mathbf {z}, \mathbf {x}) \\&\qquad \cdot \left( 1-\frac{U(\mathbf {x})}{U(\mathbf {y})}\right) \left( 1-\frac{U(\mathbf {y})}{U(\mathbf {z})}\right) \left( 1-\frac{U(\mathbf {z})}{U(\mathbf {x})}\right) . \end{aligned}\nonumber \\ \end{aligned}$$

In particular, the first equality shows that the index (2.16) is translation invariant.

A second crucial ingredient of the proof of Proposition 2.13 is the Connes area formula [18, Lemma 9.2] regarding the area of the oriented triangle spanned by three points in the plane. For every $\mathbf{p},\mathbf{x},\mathbf{y}\in \mathbb {R}^2$, let $\sin ({\angle }(\mathbf{x},\mathbf{p},\mathbf{y}))$ be the sinus of the angle of view ${\angle }(\mathbf{x},\mathbf{p},\mathbf{y}) \in (-\pi ,\pi )$ from $\mathbf{p}$ of $\mathbf{x}$ relative to $\mathbf{y}$ (which is the angle formed by the segment $\mathbf{y}-\mathbf{p}$ and $\mathbf{x}-\mathbf{p}$). One can easily check that

$$\begin{aligned} \begin{aligned}&\left( 1-\frac{U(\mathbf{x}-\mathbf{p})}{U(\mathbf{y}-\mathbf{p})}\right) \left( 1-\frac{U(\mathbf{y}-\mathbf{p})}{U(\mathbf{z}-\mathbf{p})}\right) \left( 1-\frac{U(\mathbf{z}-\mathbf{p})}{U(\mathbf{x}-\mathbf{p})}\right) \\&\qquad = -2 \mathrm {i}\left( \sin {\angle }(\mathbf{x},\mathbf{p},\mathbf{y}) +\sin {\angle }(\mathbf{y},\mathbf{p},\mathbf{z}) + \sin {\angle }(\mathbf{z},\mathbf{p},\mathbf{x}) \right) =: S(\mathbf{p},\mathbf{x},\mathbf{y},\mathbf{z}) . \end{aligned} \end{aligned}$$

Then, the Connes area formula gives

$$\begin{aligned} \begin{aligned}&\int _{\mathbb {R}^2} \mathrm{d} \mathbf{p} \,\, S(\mathbf{p},\mathbf{x},\mathbf{y},\mathbf{z})&= 2 \pi \mathrm {i}(\mathbf{x}-\mathbf{y})\wedge (\mathbf{y}-\mathbf{z}) \end{aligned} \end{aligned}$$

where $\frac{1}{2} (\mathbf{x}-\mathbf{y})\wedge (\mathbf{y}-\mathbf{z})$ is the oriented area of the triangle formed by $\mathbf{x},\mathbf{y},\mathbf{z}$.

The proof of Proposition 2.13 exploits the translation invariance of the index, expressed by (A.1), jointly with the Connes area formula, following essentially the same strategy used in [29] for discrete models.

### Proof of Proposition 2.13

To shorten the notation, let $N:={\text {Index}}(P,UPU^*)$ and $N_\mathbf{p}:={\text {Index}}(P_\mathbf{p},U_\mathbf{p}PU_\mathbf{p}^*)$. Moreover, we will denote by *K* the irrelevant strictly positive constants appearing in the proof. From (A.1) we have $N_\mathbf{p}=N$ for every $\mathbf{p} \in \mathbb {R}^2$; hence,

$$\begin{aligned} \begin{aligned} N&= |\Lambda _L|^{-1} \int _{ \Lambda _L} \mathrm{d} \mathbf{p} \, N_\mathbf{p} \\&=|\Lambda _L|^{-1}\int _{\Lambda _L} \mathrm{d} \mathbf{p} \int _{\mathbb {R}^{2}} \mathrm{d} \mathbf{x}\int _{\mathbb {R}^2} \mathrm{d} \mathbf{y}\int _{\mathbb {R}^2} \mathrm{d} \mathbf{z}\, P(\mathbf{x}, \mathbf{y}) P(\mathbf{y}, \mathbf{z}) P(\mathbf{z}, \mathbf{x}) S(\mathbf{p},\mathbf{x},\mathbf{y},\mathbf{z}) \\&=: |\Lambda _L|^{-1}\int _{\Lambda _L} \mathrm{d} \mathbf{p} \int _{\mathbb {R}^{2}} \mathrm{d} \mathbf{x}\, f(\mathbf{p},\mathbf{x}). \end{aligned} \end{aligned}$$

By suitable estimates on the decay of *f*, we have that *f* is integrable in both $\mathbb {R}^2 \times \Lambda _L$ and $\Lambda _L \times \mathbb {R}^2$, see (A.3). Therefore, the proof is reduced to show that it is possible to exchange the role of the integral in $\mathbf{p}$ with the integral in $\mathbf{x}$ up to an error of order $L^{-1/2}$, which eventually goes to zero by taking the limit $L \rightarrow \infty$. More precisely, by adding and subtracting the same term, we get

$$\begin{aligned} \begin{aligned}&\int _{\Lambda _L} d\mathbf{p} \int _{\mathbb {R}^{2}} \mathrm{d} \mathbf{x}f(\mathbf{p},\mathbf{x}) = \int _{\mathbb {R}^{2}} \mathrm{d} \mathbf{p} \int _{\Lambda _L} \mathrm{d}\mathbf{x}\, f(\mathbf{p},\mathbf{x}) - \int _{\mathbb {R}^2\setminus \Lambda _L} \mathrm{d} \mathbf{p} \int _{\Lambda _L} \mathrm{d} \mathbf{x}\, f(\mathbf{p},\mathbf{x}) \\&\quad + \int _{\Lambda _L} d\mathbf{p} \int _{\mathbb {R}^2 \setminus \Lambda _L} \mathrm{d} \mathbf{x}f(\mathbf{p},\mathbf{x}) =:\int _{\Lambda _L} \mathrm{d}\mathbf{x}\, \int _{\mathbb {R}^{2}} \mathrm{d} \mathbf{p} \, f(\mathbf{p},\mathbf{x}) + R_1 + R_2 . \end{aligned} \end{aligned}$$

By using the exponential localization of the integral kernel of *P*, we now prove a decay estimate on *f*; after that, exploiting such estimate, we show that the error terms $R_1$ and $R_2$ are of actually of order $L\sqrt{L}$.

First, by using the trivial inequality $|S(\mathbf{p},\mathbf{x},\mathbf{y},\mathbf{z})|\le 3$ and the exponential localization of the integral kernel of the projection, we get that *f* is uniformly bounded, that is $|f(\mathbf{p},\mathbf{x})|\le K$, for all $\mathbf{p},\mathbf{x}\in \mathbb {R}^2$.

Assume now that $|\mathbf{x}-\mathbf{p}|\ge 1$. We first consider the contribution to the integral in case either $\mathbf{y}$ or $\mathbf{z}$ are outside the ball of radius $|\mathbf{p}-\mathbf{x}|$ centered in $\mathbf{x}$, denoted by $B_{|\mathbf{p}-\mathbf{x}|}(\mathbf{x})$. We can bound such contribution by

$$\begin{aligned} \begin{aligned}&3 \int _{\mathbf{y}\notin B_{|\mathbf{p}-\mathbf{x}|}(\mathbf{x}) } \mathrm{d} \mathbf{y}\int _{\mathbb {R}^2} \mathrm{d} \mathbf{z}|P(\mathbf{x}, \mathbf{y}) P(\mathbf{y}, \mathbf{z}) P(\mathbf{z}, \mathbf{x})| \\&\qquad + 3 \int _{ \mathbb {R}^2 } \mathrm{d} \mathbf{y}\int _{\mathbf{z}\notin B_{|\mathbf{p}-\mathbf{x}|}(\mathbf{x})} \mathrm{d} \mathbf{z}|P(\mathbf{x}, \mathbf{y}) P(\mathbf{y}, \mathbf{z}) P(\mathbf{z}, \mathbf{x})| \\&\quad \le K_1 \int _{|\mathbf{y}-\mathbf{x}| \ge |\mathbf{p}-\mathbf{x}|} \mathrm{d} \mathbf{y}|P(\mathbf{x},\mathbf{y})| \le K \mathrm {e}^{-\frac{\beta }{2} |\mathbf{p}-\mathbf{x}|} \end{aligned} \end{aligned}$$

where we have used that *P* has an exponentially localized integral kernel. It remains to control the contribution coming from the points where both $\mathbf{y}$ and $\mathbf{z}$ are inside $B_{|\mathbf{p}-\mathbf{x}|}(\mathbf{x})$. By a geometric argument, one can prove that $\angle (\mathbf{y},\mathbf{p},\mathbf{x})$ and $\angle (\mathbf{z},\mathbf{p},\mathbf{x})$ must be in $(-\frac{\pi }{2},\frac{\pi }{2})$. Then, consider the following two estimates: for $\alpha ,\beta \in (-\frac{\pi }{2},\frac{\pi }{2})$

$$\begin{aligned} \begin{aligned} |\sin (\alpha )+\sin (\beta )-\sin (\alpha +\beta )|&= |\sin (\alpha )(1-\cos (\beta ))+\sin (\beta )(1-\cos (\alpha ))| \\&\le |\sin (\alpha )|^2|\sin (\beta )| + |\sin (\beta )|^2|\sin (\alpha )| \end{aligned} \end{aligned}$$

and, for every $\mathbf{y}$ such that $|\mathbf{y}-\mathbf{x}| < |\mathbf{p}-\mathbf{x}|$, we obtain $|\sin (\angle (\mathbf{y},\mathbf{p},\mathbf{x}))| \le |\mathbf{y}-\mathbf{x}||\mathbf{p}-\mathbf{x}|^{-1}$. Therefore, we get

A.2 $$\begin{aligned} |S(\mathbf{p},\mathbf{x},\mathbf{y},\mathbf{z})| \le \frac{|\mathbf{y}-\mathbf{x}|^2|\mathbf{x}-\mathbf{z}| + |\mathbf{y}-\mathbf{x}||\mathbf{x}-\mathbf{z}|^2 }{|\mathbf{p}-\mathbf{x}|^3} . \end{aligned}$$

Hence, by using (A.2) together with the exponential localization of *P*, we obtain

$$\begin{aligned} \begin{aligned} |f(\mathbf{p},\mathbf{x})| \le K \frac{1}{|\mathbf{x}-\mathbf{p}|^3} \int _{\mathbb {R}^2} \mathrm{d} \mathbf{y}\int _{\mathbb {R}^2} \mathrm{d} \mathbf{z}\, \mathrm {e}^{-\frac{\beta }{2}|\mathbf{x}-\mathbf{y}|} \mathrm {e}^{-\frac{\beta }{2}|\mathbf{z}-\mathbf{x}|} \le K \frac{1}{|\mathbf{x}-\mathbf{p}|^3} . \end{aligned} \end{aligned}$$

For $|\mathbf{x}-\mathbf{p}|\ge 1$, we have $\frac{\langle |\mathbf{x}-\mathbf{p}|\rangle }{|\mathbf{x}-\mathbf{p}|} \le 2$. Therefore, putting together all the previous estimates, we obtain

A.3 $$\begin{aligned} |f(\mathbf{p},\mathbf{x})|\le K_2 \chi _{|\mathbf{x}-\mathbf{p}|\le 1}(\mathbf{x},\mathbf{p}) + K_3 \chi _{|\mathbf{x}-\mathbf{p}|> 1}(\mathbf{x},\mathbf{p}) \langle \mathbf{x}-\mathbf{p} \rangle ^{-3} \le K \langle \mathbf{x}-\mathbf{p} \rangle ^{-3} \, .\nonumber \\ \end{aligned}$$

By simple estimates and integration exploiting (A.3), one can show that the error terms are of order $L\sqrt{L}$ as $L \rightarrow \infty$; thus, we obtain

$$\begin{aligned} \begin{aligned}&\int _{\Lambda _L} d\mathbf{p} \int _{\mathbb {R}^{2}} \mathrm{d} \mathbf{x}\int _{\mathbb {R}^2} \mathrm{d} \mathbf{y}\int _{\mathbb {R}^2} d z P(\mathbf{x}, \mathbf{y}) P(\mathbf{y}, \mathbf{z}) P(\mathbf{z}, \mathbf{x}) S(\mathbf{p},\mathbf{x},\mathbf{y},\mathbf{z}) \\&\quad =\int _{\Lambda _L} \mathrm{d}\mathbf{x}\int _{\mathbb {R}^{2}} d\mathbf{p} \int _{\mathbb {R}^2} \mathrm{d} \mathbf{y}\int _{\mathbb {R}^2} \mathrm{d} \mathbf{z}P(\mathbf{x}, \mathbf{y}) P(\mathbf{y}, \mathbf{z}) P(\mathbf{z}, \mathbf{x}) S(\mathbf{p},\mathbf{x},\mathbf{y},\mathbf{z}) + \mathcal {O}(L\sqrt{L}) . \end{aligned} \end{aligned}$$

Finally, by performing first the integral with respect to $\mathbf{p}$ and then using Connes area formula the proof is concluded. $\square$

## Appendix B. Technical Results

### B.1. Generalized Maclaurin–Cauchy Test

The proof of Theorem 3.1 is based on the estimates of series evaluated on points of the discrete set $\mathfrak {D}$. Because of that, it is useful to have an efficient tool to estimate the value of the series we are interested in. The next lemma concerns a generalized Maclaurin–Cauchy estimate that serves exactly this purpose.

#### Lemma B.1

(Generalized Maclaurin–Cauchy test). Let $\mathfrak {D}\subset \mathbb {R}^2$ be a *r*-uniformly discrete set. Consider a continuous function

$$\begin{aligned} D: \mathbb {R}^2 \rightarrow \mathbb {R}\, , \end{aligned}$$

such that $|D(\mathbf{x})| \ge |D(\mathbf{y})|$ whenever $|\mathbf{x}|\le |\mathbf{y}|$. Then, there exists a constant $K_r$, depending on *r* but independent of *L*, such that for every $L>2r$ it holds that

B.1 $$\begin{aligned} \sum _{\gamma \in \mathfrak {D}\cap \Lambda _L } |D(\gamma ) | \le K_r \int _{\Lambda _L} \mathrm{d}\mathbf{x}|D(\mathbf{x})| \, . \end{aligned}$$

#### Proof

The proof is based on the same argument of the well-known Maclaurin–Cauchy integral test. First of all, by the hypothesis on $\mathfrak {D}$, it holds that

$$\begin{aligned} \mathfrak {D}\cap B_{r}(\gamma ) = \left\{ \gamma \right\} \; , \qquad \forall \gamma \in \mathfrak {D}. \end{aligned}$$

Fix $\rho =2r$. By hypothesis on $\mathfrak {D}$, the number of points of $\mathfrak {D}$ such that $|\gamma |<\rho$ is finite, so their contribution to the series is finite, say $K_\rho \in \mathbb {R}_+$. Hence, one has

B.2 $$\begin{aligned} \sum _{\gamma \in \mathfrak {D}\cap \Lambda _L} |D(\gamma ) |= K_\rho + \sum _{\gamma \in \mathfrak {D}\cap \Lambda _L, |\gamma |\ge \rho } |D(\gamma ) |\, . \end{aligned}$$

For every point $\gamma \in \mathfrak {D}\cap \Lambda _L$ such that $|\gamma | \ge \rho$, one constructs a square $A_r(\gamma )$ of area $\frac{r^2}{2}$ such that one of its vertices is $\gamma$ and for all $\mathbf{x}\in A_r(\gamma )$ it holds that $|\mathbf{x}| \le |\gamma |$. (For example, $A_r(\gamma )$ might be the open square of diagonal length equal to *r* constructed along the line passing through the origin and $\gamma$). It is also true that

$$\begin{aligned} A_r(\gamma ) \cap \mathfrak {D}= \left\{ \gamma \right\} \, , \qquad A_r(\gamma ) \subset \Lambda _L \, . \end{aligned}$$

Therefore, we obtain that

$$\begin{aligned} \sum _{\gamma \in \mathfrak {D}\cap \Lambda _L, |\gamma |\ge \rho } |D(\gamma ) |&= \frac{2}{r^2}\sum _{\gamma \in \mathfrak {D}\cap \Lambda _L, |\gamma |\ge \rho } |D(\gamma ) | \frac{r^2}{2} \\&\le \frac{2}{r^2} \sum _{\gamma \in \mathfrak {D}\cap \Lambda _L, |\gamma |\ge \rho } \int _{ A_r(\gamma )} \; \mathrm{d}\mathbf{x}\,\, |D(\mathbf{x})| \le \frac{2}{r^2} \int _{ \Lambda _L} \mathrm{d} \mathbf{x}|D(\mathbf{x})| \, . \end{aligned}$$

Then, considering (B.2) and $\Lambda _\rho \subseteq \Lambda _L$, we get that

B.3 $$\begin{aligned} \sum _{\gamma \in \mathfrak {D}\cap \Lambda _L} |D(\gamma ) |\le \left( \frac{r^2 K_\rho }{2}\left( \int _{ \Lambda _\rho } \mathrm{d} \mathbf{x}\, |D(\mathbf{x})|\right) ^{-1} + 1\right) \frac{2}{r^2} \int _{ \Lambda _L} \mathrm{d} \mathbf{x}|D(\mathbf{x})| \, .\nonumber \\ \end{aligned}$$

This proves the claim, with the constant $K_r$ given by the bracketed expression exhibited in the l.h.s of (B.3). $\square$

#### Remark B.2

The result of Lemma B.1, namely inequality (B.1), still holds true if, instead of the radial monotonicity of the function $D: \mathbb {R}^2 \rightarrow \mathbb {R}$, we require only a directional monotonicity, that is $|D(\mathbf{x})| \ge |D(\mathbf{y})|$ whenever $|\mathbf{x}_i|\le |\mathbf{y}_i|$, for $i=1$ or $i=2$. The strategy of the proof is exactly the same, one just need to replace the square $A_r(\gamma )$ with another suitable *r*-dependent set such that $A_r(\gamma ) \subset \Lambda _L$, $|A_r(\gamma )|=\frac{r^2}{2}$, and $|\mathbf{x}_i|\le |\gamma _i|$ for all $\mathbf{x}\in A_r(\gamma )$.

### B.2. Properties of the Operators $\Gamma _i$

In this section, we collect the properties of the operators $\Gamma _i$ that are used in the proof of Theorem 3.1.

#### Lemma B.3

Let $\mathfrak {D}$ be a *r*-uniformly discrete set, and $\left\{ \psi _{\gamma , a} \right\} _{\gamma \in \mathfrak {D}, 1 \le a \le m(\gamma )}$ a GWB *G*-localized around $\mathfrak {D}$. Then, the operators $\Gamma _i$ defined by (4.3) are integral operators. Moreover,

(i) if $G(\Vert \mathbf{x}\Vert )={\mathrm {e}}^{2\alpha \Vert \mathbf{x}\Vert }$ with $\alpha <\beta$ for $\beta$ as in (2.2), then the integral kernel $\Gamma _i(\mathbf{x},\mathbf{y})$ satisfies
  $$\begin{aligned} \left| \Gamma _i(\mathbf{x},\mathbf{y}) \right| \le C {\mathrm {e}}^{-\beta ' |\mathbf{x}-\mathbf{y}|} + C' |x_i| {{\,\mathrm{e}\,}}^{-\beta ' | \mathbf{x}-\mathbf{y}|} \end{aligned}$$
  for $$C,C' >0$$ and $$\beta '<\alpha $$.
(ii) if $G(\Vert \mathbf{x}\Vert )=\langle \mathbf{x}\rangle ^{2s}$ with $s>\frac{3}{2}$, then the integral kernel $\Gamma _i(\mathbf{x},\mathbf{y})$ satisfies
  $$\begin{aligned} \left| \Gamma _i(\mathbf{x},\mathbf{y}) \right| \le C \langle \mathbf{x}-\mathbf{y}\rangle ^{-(s-\frac{3}{2}-\epsilon )} + C' |x_i| \, \langle \mathbf{x}- \mathbf{y}\rangle ^{-(s-1-\epsilon )} \end{aligned}$$
  for every $$0<\epsilon <s-\frac{3}{2}$$.

#### Proof

Let us show only the proof of (ii), since the proof of (i) is similar and simpler. The formal integral kernel of $\Gamma _i$ is given by

$$\begin{aligned} \Gamma _i(\mathbf{x},\mathbf{y})= \sum _{\gamma ,a} \gamma _i \psi _{\gamma ,a}(\mathbf{x}) \overline{\psi _{\gamma ,a}}(\mathbf{y}) \, . \end{aligned}$$

For every fixed $\mathbf{x},\mathbf{y}\in \mathbb {R}^2$, the sum over the indices $\{\gamma ,a\}$ is absolutely convergent. Indeed, fix $0<\epsilon <s- \frac{3}{2}$, since $\left\| \mathbf{x}-\gamma \right\| \langle \mathbf{x}- \gamma \rangle ^{-1} \le 1$, we get that

B.4 $$\begin{aligned} \begin{aligned} \left| \Gamma _i(\mathbf{x},\mathbf{y}) \right|&\le \sum _{\gamma ,a} \langle \mathbf{x}- \gamma \rangle ^{-(s-1)} \langle \mathbf{y}- \gamma \rangle ^{-s} + \sum _{\gamma ,a} |x_i| \langle \mathbf{x}- \gamma \rangle ^{-s} \langle \mathbf{y}- \gamma \rangle ^{-s} \\&\le C_s \langle \mathbf{x}- \mathbf{y}\rangle ^{-(s-\frac{3}{2}-\epsilon )} \sum _{\gamma ,a} \langle \mathbf{y}- \gamma \rangle ^{-(\frac{3}{2}+\epsilon )} \langle \mathbf{x}- \gamma \rangle ^{-(\frac{1}{2}+\epsilon )} \\&\qquad + C_s |x_i| \, \langle \mathbf{x}- \mathbf{y}\rangle ^{-(s-1-\epsilon )} \sum _{\gamma ,a} \langle \mathbf{y}- \gamma \rangle ^{-(1+\epsilon )} \langle \mathbf{x}- \gamma \rangle ^{-(1+\epsilon )} \\&\le C \langle \mathbf{x}-\mathbf{y}\rangle ^{-(s-\frac{3}{2}-\epsilon )} + C' |x_i| \, \langle \mathbf{x}- \mathbf{y}\rangle ^{-(s-1-\epsilon )} \, , \end{aligned}\nonumber \\ \end{aligned}$$

where again in the first inequality we have used $L^\infty$ estimate on the GWF (2.6), in the second inequality we have used the property (2.1) of the localization function *G* (denoting by $C_s$ the constant $C_G$ appearing in (2.1)) and in the last inequality we have used Hölder’s inequality. Hence, the operators $\Gamma _i$ admit an integral kernel. $\square$

As a consequence of the previous estimates on the integral kernels, the operators $\Gamma _i$ enjoy some trace class properties.

#### Proposition B.4

Let *P* be an exponentially localized projection and $X_i$, $\Gamma _i$, for $i\in \left\{ 1,2\right\}$ and $\chi _{\Lambda _L}$ as defined above. Assume that *P* admits a GWB, *G*-localized around a *r*-uniformly discrete set $\mathfrak {D}$, with localization function $G(\Vert \mathbf{x}\Vert ) = \langle \mathbf{x}\rangle ^{2s}$ for some $s>\frac{7}{2}$. Then, for every $i,j \in \{1,2\}$ the operators

$$\begin{aligned} \chi _{\Lambda _L} P X_i P \; \Gamma _j \; \; , \chi _{\Lambda _L} P \; \Gamma _i P \; X_j \; \; , \chi _{\Lambda _L} P \; \Gamma _i P \; \Gamma _j \; , \end{aligned}$$

are trace class in $L^2(\mathbb {R}^2)$.

#### Proof

The strategy of the proof is the same of Proposition 4.1. Let us show the computations explicitly for the operator $\chi _{\Lambda _L}\; P \;\Gamma _1 \; P \; X_2$, the other cases can be treated similarly.

Since $\Gamma _1$ commutes with *P*, and $P^2=P$, we have that

$$\begin{aligned} \begin{aligned}&\chi _{\Lambda _L}\; P \;\Gamma _1 \; P \; X_2 = \chi _{\Lambda _L}\; P \;P \;\Gamma _1 \; X_2 = \chi _{\Lambda _L}\; P \;\Gamma _1 \; X_2 \; \\&\qquad = \chi _{\Lambda _L}\; {\mathrm {e}}^{2\alpha \Vert X\Vert }\; {\mathrm {e}}^{-2\alpha \Vert X\Vert } \;P\; {\mathrm {e}}^{\alpha \Vert X\Vert }\; {\mathrm {e}}^{-\alpha \Vert X\Vert }\; \Gamma _1 \; \langle X \rangle \; \langle X \rangle ^{-1} \; X_2 \, , \end{aligned} \end{aligned}$$

where we have chosen the constant $\alpha$ strictly smaller than the exponent $\beta$ appearing in (2.2). Then, by using the estimate (2.2) and triangular inequality, we have that

$$\begin{aligned} \left| \left( {\mathrm {e}}^{-2\alpha \Vert X\Vert }\; P \; {\mathrm {e}}^{\alpha \Vert X\Vert } \right) (\mathbf{x},\mathbf{y}) \right| \le C {\mathrm {e}}^{-\alpha \Vert \mathbf{x}\Vert } {\mathrm {e}}^{-(\beta -\alpha ) \Vert \mathbf{x}-\mathbf{y}\Vert } \end{aligned}$$

for some positive constants *C*. Therefore, $\left( {\mathrm {e}}^{-2\alpha \Vert X\Vert }\; P \; {\mathrm {e}}^{\alpha \Vert X\Vert } \right)$ is a Hilbert–Schmidt operator. Similarly, considering (B.4) instead of (2.2), we have

$$\begin{aligned} \left| \left( {\mathrm {e}}^{-\alpha \Vert X\Vert } \;\Gamma _1\; \langle X \rangle \right) (\mathbf{x},\mathbf{y}) \right| \le {\mathrm {e}}^{-\tilde{\alpha } \Vert \mathbf{x}\Vert } C \langle \mathbf{x}-\mathbf{y}\rangle ^{-(s-\frac{5}{2}-\epsilon )} \,, \end{aligned}$$

where $0<\tilde{\alpha }<\alpha$. Since $s>\frac{7}{2}$, we can choose $\epsilon$ small enough so that the integral kernel of $\left( {\mathrm {e}}^{-\alpha \Vert X\Vert } \;\Gamma _1\; \langle X \rangle \right)$ is in $L^2(\mathbb {R}^2\times \mathbb {R}^2)$ and hence the operator is Hilbert–Schmidt. Eventually, since $\langle X \rangle ^{-1} \; X_2$ and $\chi _{\Lambda _L}\; {\mathrm {e}}^{2\alpha \Vert X\Vert }$ are bounded operators and the product of two Hilbert–Schmidt operators is trace class, the proof is over. $\square$

#### Remark B.5

Although the proofs of Proposition 4.1 and Proposition B.4 appear similar, their hypotheses have a crucial difference. Proposition 4.1 requires only the localization of the integral kernel of the projection, which follows from the existence of a gap in the spectrum of *H*, while Proposition B.4 requires, beyond the gap assumption, the existence of a GWB for the projection with a particular decay of the GWFs.

### B.3. Proof of Proposition 4.2

Consider $\Lambda _L = [-L,L]^2 \subset \mathbb {R}^2$ and $\gamma \in \mathfrak {D}\cap \Lambda _L$. To estimate “how much of $\psi _{\gamma ,a}$ is outside $\Lambda _L$,” we consider $\left\| \chi _{\Lambda ^c_L} \psi _{\gamma ,a} \right\|$ where $\chi _{\Lambda _L^c}$ is the characteristic function of the complementary set of $\Lambda _L$. We get

B.5 $$\begin{aligned} \begin{aligned} \left\| \chi _{\Lambda _L^c}\psi _{\gamma ,a} \right\|&\le \left( \sup _{\mathbf{x}\in \mathbb {R}^2} \left( \chi _{\Lambda _L^c}(\mathbf{x}) \langle \mathbf{x}-\gamma \rangle ^{-2s} \right) \int _{\mathbb {R}^2} \langle \mathbf{x}-\gamma \rangle ^{2s} |\psi _{\gamma ,a}(\mathbf{x})|^2 \mathrm{d}\mathbf{x}\right) ^{\frac{1}{2}} \\&\le M^{\frac{1}{2}}\left( \langle L-|\gamma _1|\rangle ^{-2s} + \langle L-|\gamma _2|\rangle ^{-2s}\right) \, . \end{aligned}\nonumber \\ \end{aligned}$$

Using Remark B.2, one can see by explicit integration that, if $s>\frac{1}{2}$, then both terms on the right-hand side of (B.5) are integrable in $\mathbb {R}$ and therefore there exists a positive constant $\mathcal {I}_{\textsc {mo}}$ such that $\sum \limits _{\xi \in \Lambda _L , \, c} \left\| \chi _{\Lambda _L^c} \psi _{\xi ,c}\right\| \le \mathcal {I}_{\textsc {mo}}L$ .

If, instead, $\gamma$ is outside $\Lambda _L$ we have that

B.6 $$\begin{aligned} \begin{aligned} \left\| \chi _{\Lambda _L} \psi _{\gamma ,a} \right\|&\le \left( \sup _{\mathbf{x}\in \mathbb {R}^2} \left[ \chi _{\Lambda _L}(\mathbf{x}) \langle \mathbf{x}-\gamma \rangle ^{-2s}\right] \int _{\mathbb {R}^2} \langle \mathbf{x}-\gamma \rangle ^{2s} |\psi _{\gamma ,a}(\mathbf{x})|^2 \mathrm{d}\mathbf{x}\right) ^{\frac{1}{2}} \\&\le M^{\frac{1}{2}}\left( \langle |\gamma _1|-L\rangle ^{-2s}\chi _{A_1}(\gamma ) + \langle |\gamma _2|-L\rangle ^{-2s}\chi _{A_2}(\gamma ) \right) \\&\qquad + M^{\frac{1}{2}}\langle \sqrt{||\gamma _1|-L|^2 +||\gamma _2|-L|^2}\rangle ^{-2s} \chi _{A_3}(\gamma ) \,, \end{aligned} \end{aligned}$$

where $A_1:=([-\infty ,-L]\cup [L,\infty ])\times [-L,L]$, $A_2:= [-L,L]\times ([-\infty ,-L]\cup [L,\infty ])$ and $A_3:=\Lambda _L^c \setminus (A_1 \cup A_2)$, and $\chi _{A_i}$, with $i \in \left\{ 1,2,3\right\}$, is the characteristic function of the set $A_i$. As in the previous case, using Lemma B.1 and Remark B.2, one can see by explicit integration that, if $s>\frac{1}{2}$, then the first two terms on the right-hand side of (B.6) are integrable in $\mathbb {R}$ and, if $s>1$, the last term is integrable in $\mathbb {R}^2$. Therefore, there exists a positive constant $\mathcal {I}_{\textsc {mi}}$ such that $\sum \nolimits _{\xi \notin \Lambda _L \, , c} \Vert \chi _{\Lambda _L} \psi _{\xi ,c}\Vert \le \mathcal {I}_{\textsc {mi}}L$ .

It remains to prove the second part of Proposition 4.2, concerning the off-diagonal terms of $\widetilde{X}_i$. Considering the generic matrix element of $\widetilde{X}_1-\Gamma _1$, one has

$$\begin{aligned}&\sum \limits _{a=1}^{m(\gamma )}\sum \limits _{b=1}^{m(\eta )}|\left\langle \psi _{\gamma ,a}, (X_1-\gamma _1)\psi _{\eta ,b} \right\rangle |\\&\qquad \le (m_*)^2 \max _{1\le a \le m(\gamma )} \max _{ 1\le b \le m(\eta )}|\left\langle \psi _{\gamma ,a}, (X_1-\gamma _1) \psi _{\eta ,b} \right\rangle | \\&\qquad \le (m_*)^2 \int _{\mathbb {R}^2} \mathrm{d}\mathbf{x}\, \left| \psi _{\gamma ,{\tilde{a}}}(\mathbf{x})\right| |x_1-\gamma _1| \left| \psi _{\eta ,{\tilde{b}}}(\mathbf{x})\right| , \end{aligned}$$

where ${\tilde{a}}$ and ${\tilde{b}}$ are the maximizers of $|\left\langle \psi _{\gamma ,a}, (X_1-\gamma _1) \psi _{\eta ,b} \right\rangle |$. Therefore, we get that

$$\begin{aligned}&\langle \gamma - \eta \rangle ^{s-1} (m_*)^2 \int _{\mathbb {R}^2} \mathrm{d}\mathbf{x}\,\left| \psi _{\gamma ,{\tilde{a}}}(\mathbf{x}) \right| \, |x_1-\gamma _1| \left| \psi _{\eta ,{\tilde{b}}}(\mathbf{x}) \right| \\&\qquad \le C_s (m_*)^2 \int _{\mathbb {R}^2} \mathrm{d}\mathbf{x}\,\langle \gamma - \mathbf{x}\rangle ^{s-1} \left| \psi _{\gamma ,{\tilde{a}}}(\mathbf{x}) \right| \langle \mathbf{x}-\gamma \rangle \langle \mathbf{x}- \eta \rangle ^{s-1} \left| \psi _{\eta ,{\tilde{b}}}(\mathbf{x}) \right| \\&\qquad \le C_s (m_*)^2 M \, , \end{aligned}$$

where in the last inequality we have used Cauchy–Schwarz inequality together with property (2.5). This implies that

$$\begin{aligned} (m_*)^2 \int _{\mathbb {R}^2} \mathrm{d}\mathbf{x}\,\left| \psi _{\gamma ,a}(\mathbf{x}) \right| \, |x_1-\gamma _1| \left| \psi _{\eta ,{b}}(\mathbf{x}) \right| \le k_s \langle \gamma - \eta \rangle ^{-(s-1)}. \end{aligned}$$

As clear from the above computation, $k_s$ depends, actually, also on $m_*, K, C_s$. The same computation goes through exchanging $X_1-\gamma _1$ with $X_2-\gamma _2$. Therefore, the function *F* defined by

B.7 $$\begin{aligned} F(\Vert \mathbf{x}\Vert ):= k_s \langle \mathbf{x}\rangle ^{-(s-1-\epsilon )} \end{aligned}$$

satisfies (4.7). A direct computation shows that *F* satisfies also the requirements (4.8) for every $s>3$, (4.9) for every $s>2$, and (4.10) for every $s>4$.

Finally, we show that (4.11) is satisfied for every $s>3$. Indeed, let $\epsilon =\frac{s-3}{100}$ and consider the integral

B.8 $$\begin{aligned} \int _{\Lambda _L} \mathrm{d} \mathbf{x}\int _{\mathbb {R}^2\setminus \Lambda _L} \mathrm{d}\mathbf{y}\, \frac{1}{(1+ \Vert \mathbf{x}-\mathbf{y}\Vert ^2)^{(s-1-\epsilon )}} \, . \end{aligned}$$

Since the integrand is positive, the order of integration does not affect the result. For a fixed $\mathbf{x}\in \Lambda _L$ and $\alpha :=s-1-\epsilon$, we have the inequality

B.9 $$\begin{aligned} \frac{1}{(1+ \Vert \mathbf{x}-\mathbf{y}\Vert ^2)^\alpha } \le \frac{1}{(1+ \Vert \mathbf{x}-\mathbf{y}\Vert ^2)^\frac{\alpha }{2}} \frac{1}{(1+ ({{\,\mathrm{dist}\,}}(\mathbf{x},\partial \Lambda _L))^2)^\frac{\alpha }{2}} \, . \end{aligned}$$

Since $s>3$, the right-hand side in (B.9) is integrable in $\mathbf{y}$, and hence we obtain

$$\begin{aligned} \begin{aligned}&\int _{\Lambda _L} \mathrm{d} \mathbf{x}\int _{\mathbb {R}^2\setminus \Lambda _L} \mathrm{d}\mathbf{y}\, \frac{1}{(1+ \Vert \mathbf{x}-\mathbf{y}\Vert ^2)^\alpha } \le C \int _{\Lambda _L} \mathrm{d} \mathbf{x}\frac{1}{(1+ ({{\,\mathrm{dist}\,}}(\mathbf{x},\partial \Lambda _L))^2)^\frac{\alpha }{2}} \end{aligned} \end{aligned}$$

for some positive constant *C*. By an elementary estimate and explicit integration, one can see that the integral with respect to $\mathbf{x}$ is of order *L*. This concludes the proof. $\square$

## Footnotes

1 The vanishing of the numbers below expresses the vanishing of the *first Chern class* $c_1(P)$ as an element of the cohomology space $H^1(\mathbb {T}^3_*, \mathbb {R})$, which also implies the vanishing of the *integer* first Chern class in $H^1(\mathbb {T}^3_*, \mathbb {Z})$; see [72].

2 Whenever we use the adjective “generalized” we refer to Wannier functions for non-periodic systems. However, this terminology is far from universal. The reader is warned that the adjective “generalized” has been occasionally used referring to Wannier functions for a multi-band periodic system (which are called *composite* Wannier functions in the mathematical literature), as, for example, in [60].

3 In this system of units the reduced Planck constant, the electron charge and the electron mass are dimensionless and equal to 1. In particular, the quantum of charge conductivity in the quantum Hall effect is $\frac{e^2}{h}=\frac{1}{2\pi }$, where $-e$ is the electron charge and *h* is the Planck constant.

4 Recall that a Bravais lattice $\Gamma \subset \mathbb {R}^d$ is defined as a discrete subgroup of $(\mathbb {R}^d, +)$ with maximal rank. It follows that there exist a (non-unique) linear basis $\left\{ \mathbf {a}_1, \ldots , \mathbf {a}_d \right\} \subset {\mathbb {R}^d}$ such that $\Gamma = {{\,\mathrm{Span}\,}}_{\mathbb {Z}} \left\{ \mathbf {a}_1, \ldots , \mathbf {a}_d \right\} \simeq \mathbb {Z}^d$.

5 A discrete set is said to be *R*-*uniformly nowhere sparse* if there exists $R>0$ such that $\forall \, \mathbf{x}\in \mathbb {R}^d$ the set $B_{R}(\mathbf{x}) \cap \mathfrak {D}$ contains at least one element.

6 See (2.3) for the definition of $L^2_{\mathrm{{uloc}}}(\mathbb {R}^2)$, which can be easily generalized to any dimension.

7 Notice that all the terms involved in the direct computation are trace class operators, as it is proved in Proposition 4.1.

8 Here, we use the adverb “conditionally” to stress the fact that $\tau (\cdot )$ is cyclic under some additional conditions, *e.g.*, the operators involved in its arguments are periodic. Indeed, even if the Chern character can be expressed as the trace per unit volume of the commutator between the reduced position operators $\widetilde{X}_1$ and $\widetilde{X}_2$, it may very well be nonvanishing. (The reduced position operators are not periodic.)

9 In our setting, a canonical time-reversal operator is simply given by the complex conjugation operator.

10 Notice that in [29] the authors focus on the Hall conductance, which, however, for $d=2$, as in our setting, equals the Hall conductivity and hence is proportional to the Chern character.
